# Beyond the Pandemic Era: Recent Advances and Efficacy of SARS-CoV-2 Vaccines Against Emerging Variants of Concern

**DOI:** 10.3390/vaccines13040424

**Published:** 2025-04-17

**Authors:** Ankita Saha, Sounak Ghosh Roy, Richa Dwivedi, Prajna Tripathi, Kamal Kumar, Shashank Manohar Nambiar, Rajiv Pathak

**Affiliations:** 1 Department of Cell Biology, Albert Einstein College of Medicine, Bronx, New York, NY 10461, USA; ankita.saha@einsteinmed.edu; 2 Henry M. Jackson Foundation for the Advancement of Military Medicine, Naval Medical Research Command, Silver Spring, MD 20910, USA; sounak.ghoshroy.ctr@health.mil; 3 Department of Microbiology, Immunology, and Physiology, Meharry Medical College, Nashville, TN 37208, USA; rdwivedi@mmc.edu; 4 Department of Microbiology and Immunology, Weill Cornell Medical College, New York, NY 10021, USA; prt4001@med.cornell.edu; 5 Department of Cellular and Molecular Medicine, University of California at San Diego, La Jolla, CA 92093, USA; kakumar@ucsd.edu; 6 Division of Hepatology, Department of Medicine, Albert Einstein College of Medicine, Bronx, New York, NY 10461, USA; shashank.nambiar@einsteinmed.edu; 7 Department of Microbiology and Immunology, Albert Einstein College of Medicine, Bronx, New York, NY 10461, USA; 8 Department of Genetics, Albert Einstein College of Medicine, Bronx, New York, NY 10461, USA

**Keywords:** SARS-CoV-2, COVID-19, post-pandemic era, variants of concern (VOCs), Omicron subvariants, next-generation vaccines, pan-coronavirus, multivalent vaccines

## Abstract

Vaccination has been instrumental in curbing the transmission of SARS-CoV-2 and mitigating the severity of clinical manifestations associated with COVID-19. Numerous COVID-19 vaccines have been developed to this effect, including BioNTech-Pfizer and Moderna’s mRNA vaccines, as well as adenovirus vector-based vaccines such as Oxford–AstraZeneca. However, the emergence of new variants and subvariants of SARS-CoV-2, characterized by enhanced transmissibility and immune evasion, poses significant challenges to the efficacy of current vaccination strategies. In this review, we aim to comprehensively outline the landscape of emerging SARS-CoV-2 variants of concern (VOCs) and sub-lineages that have recently surfaced in the post-pandemic years. We assess the effectiveness of existing vaccines, including their booster doses, against these emerging variants and subvariants, such as BA.2-derived sub-lineages, XBB sub-lineages, and BA.2.86 (Pirola). Furthermore, we discuss the latest advancements in vaccine technology, including multivalent and pan-coronavirus approaches, along with the development of several next-generation coronavirus vaccines, such as exosome-based, virus-like particle (VLP), mucosal, and nanomaterial-based vaccines. Finally, we highlight the key challenges and critical areas for future research to address the evolving threat of SARS-CoV-2 subvariants and to develop strategies for combating the emergence of new viral threats, thereby improving preparedness for future pandemics.

## 1. Introduction

The COVID-19 pandemic, caused by severe acute respiratory syndrome coronavirus-2 (SARS-CoV-2) in late 2019, severely impacted global public health systems, disrupting healthcare infrastructure and supply chains while causing widespread illness and death. However, due to reduced fatalities and declining hospitalizations, the WHO declared on 5 May 2023 that COVID-19 is no longer a public health emergency of international concern [1]. According to the International Committee on Taxonomy of Viruses (ICTV), SARS-CoV-2 is classified within the order *Nidovirales*, suborder *Cornidovirineae*, family *Coronaviridae*, subfamily *Coronavirinae*, genus *Betacoronavirus*, and subgenus *Sarbecovirus* [2]. SARS-CoV-2 has a single-stranded, positive-sense RNA genome ranging from approximately 29.7 to 29.9 kilobases (kb) in length. This genome encodes structural, non-structural, and accessory proteins, which are essential for viral entry, replication, immune evasion, and persistence within host cells [3,4]. Viral entry is primarily mediated by the interaction between the S1 subunit of the trimeric spike (S) protein and the host angiotensin-converting enzyme 2 (ACE2) receptor, facilitating membrane fusion and subsequent infection. Since the S1 subunit is critical for viral attachment and entry, it has been a key target for vaccine development, with most SARS-CoV-2 vaccines utilizing the spike protein as the primary antigen [5]. Vaccine-induced protective immunity involves multiple arms of the immune system to eliminate the virus. The adaptive immune response, triggered by vaccination or natural infection, comprises nAbs, memory B cells, CD4^+^ T cells, and CD8^+^ T cells [6]. Among these components, nAbs play a crucial role in host protection by binding to the viral surface, preventing viral entry into host cells, and inhibiting replication, thereby mitigating disease progression [7].

Coronaviruses, including SARS-CoV-2, exhibit lower mutation rates compared to many other RNA viruses due to the presence of nonstructural protein 14 (nsp14). Nsp14 contains an exoribonuclease (ExoN) domain that provides a proofreading function during RNA replication, thereby enhancing replication fidelity. Despite this proofreading capability, SARS-CoV-2 can still accumulate mutations due to replication errors by its RNA-dependent RNA polymerase (RdRp) and selective pressures within the host environment, such as immune responses and antiviral treatments [8,9]. The emergence of new SARS-CoV-2 variants of concern (VOCs) is driven by genetic changes that may confer advantages in viral fitness, transmissibility, pathogenicity, and/or immune evasion. Notably, all identified SARS-CoV-2 VOCs possess mutations in the spike (S) protein, particularly within the receptor-binding domain (RBD), which may enhance ACE2 binding affinity or facilitate immune escape [10]. Such mutations can alter interactions with neutralizing antibodies (nAbs), potentially reducing immune recognition and impacting vaccine efficacy (VE). The initial SARS-CoV-2 vaccines developed using the original Wuhan B.1 strain effectively elicited neutralizing responses against the parental strain. However, their neutralization potency significantly declined against VOCs. Sera from individuals vaccinated with these first-generation vaccines demonstrated reduced neutralization capacity against nearly all VOCs, including Alpha, Beta, Gamma, and Omicron [11,12,13,14,15]. The inability to fully contain SARS-CoV-2 and its continued circulation has driven the emergence of multiple sub-lineages, including XBB and BA.2.86, which exhibit enhanced transmissibility, infectivity, and immune evasion properties [16,17,18]. The decreased VE against XBB variants correlates with acquired mutations in the RBD of the spike protein, such as F486P and F456L, both of which confer immune escape from nAbs [19]. Notably, BA.2.86 demonstrates stronger binding affinity to the ACE2 receptor compared to other variants [20,21,22]. Further evolution within the XBB and BA.2.86 lineages has led to the emergence of sub-lineages with additional spike protein substitutions, including XBB.1.5 (S486P in XBB) and JN.1 (L455S in BA.2.86). In 2023 and 2024, the global SARS-CoV-2 landscape was dominated by sub-lineages derived from XBB (XBB.1.5, XBB.1.9.1, XBB.1.16.1, EG.5.1.1, EG.5.1.3, XBF, BA.2.86.1, EG.5-like, FL1.5.1-like, HV.1, and HK.3) and JN.1 (KQ.1-like, KP.2-like, KP.3, LB.1-like, and KP3.1.1) [23].

The continuous evolution of the antigenic landscape of SARS-CoV-2 necessitates the development of an efficient, broad-spectrum vaccine candidate that provides long-lasting protection to effectively control the disease. Several monovalent and bivalent vaccines have been developed using VOCs as antigens. A monovalent vaccine targets a single strain, whereas a bivalent vaccine incorporates antigens from two different SARS-CoV-2 strains. However, as new vaccines are developed using the most recent VOCs, additional VOCs continue to emerge, often reducing the efficacy of the existing vaccines. For instance, Moderna has developed multiple vaccines incorporating different SARS-CoV-2 strains as antigens: (1) the original Wuhan B.1 strain (monovalent), (2) a bivalent vaccine targeting Wuhan B.1 and Omicron BA.1, (3) a bivalent vaccine targeting Wuhan B.1 and Omicron BA.4/BA.5, and (4) the most recent monovalent vaccine based on the Omicron KP.2 strain (2024–2025 formula). Notably, the first three vaccines are no longer authorized for use in the United States due to their reduced effectiveness against newly emerged VOCs [24]. Similarly, multiple biotech and pharmaceutical industries have developed vaccines targeting previous VOCs, but the rapid evolution of SARS-CoV-2 continues to pose challenges. The most recent SARS-CoV-2 vaccines authorized by the U.S. FDA include the Moderna and Pfizer COVID-19 monovalent vaccines formulated with the Omicron KP.2 strain (2024–2025 formula) and the Novavax COVID-19 monovalent vaccine based on the Omicron JN.1 strain, adjuvanted (2024–2025 formula) [24]. However, like their predecessors, these updated vaccines may not be optimal in controlling SARS-CoV-2 transmissibility and infectivity, as they follow the same antigenic selection strategy. Over time, the neutralizing capacity of these vaccines is likely to diminish against newly emerging VOCs, necessitating continuous updates and adaptations to maintain protective efficacy.

Apart from vaccines, several monoclonal antibodies (mAbs) have been developed for therapeutic use against SARS-CoV-2. However, due to the continuous evolution of the virus and the accumulation of mutations in the spike protein, many of these mAbs have become less effective against newly emerging VOCs [15]. An alternative approach to address the emergence of new VOCs is the development of recombinant vaccines by incorporating mutations from multiple SARS-CoV-2 strains within a single polypeptide, serving as a broad-spectrum antigen. Recently, Herwig and colleagues explored the strategy of merging spike (S) protein mutations from different VOCs into a single protein, developing a vaccine, and evaluating its immune protection by assessing the neutralizing potential of antibodies [25].

The primary driver of VOC emergence is the transmission of the virus between individuals, which predominantly occurs via the upper respiratory tract. The parental SARS-CoV-2 vaccines, administered intramuscularly (IM), have been shown to elicit suboptimal immunity at the upper respiratory tract mucosa [26,27,28]. To enhance both systemic and mucosal immunity, multiple intranasal vaccines (NVs) have been developed and tested in various model systems [29,30,31,32]. Similar to IM vaccines, NVs stimulate cellular, mucosal, and humoral immune responses [33]. A combined approach using both IM and NV formulations has demonstrated improved mucosal immunity compared to IM vaccines alone [34,35,36]. To extend protection and address waning immunity in vaccinated individuals, self-amplifying mRNA (SA-mRNA) vaccines have been developed and are currently under clinical trials [37,38]. The overarching challenge remains the development of a universal SARS-CoV-2 vaccine capable of providing broad protection against all VOCs. Potential strategies to achieve this goal have been discussed by Moore and colleagues [39].

This review provides a comprehensive analysis of the SARS-CoV-2 variants and sub-lineages that have emerged during the pre- and post-pandemic periods, along with recent advancements in vaccine development and their efficacy against newly identified VOCs. Emphasis is placed on BA.2-derived sub-lineages (BJ.1 and BM.1.1.1), XBB sub-lineages (XBB.1.9, XBB.1.5, and XBB.1.16), JN.1, and BA.2.86 (Pirola). Furthermore, we examine recent advancements in vaccine technology aimed at enhancing both the breadth and durability of immune protection. These include multivalent vaccine formulations and next-generation SARS-CoV-2 vaccines strategies designed to mitigate the impact of ongoing viral evolution. Additionally, we evaluate progress in vaccine platforms—such as mRNA, vector-based, and protein subunit vaccines—that have demonstrated the potential to elicit robust and long-lasting immune responses. Strategies to broaden immunological coverage are also discussed, including optimized antigen design, novel adjuvants, and innovative delivery systems. Lastly, we discuss the critical challenges and lessons learned from the SARS-CoV-2 pandemic, which will help strengthen vaccine development efforts, enhance public health preparedness, and improve global response strategies for future outbreaks of emerging viral infections.

## 2. SARS-CoV-2 Variants of Concern, Evolution, and Impact on Public Health

SARS-CoV-2 continues to evolve under selective pressures from host immunity, vaccination, and antiviral treatments, leading to the emergence of VOCs with enhanced transmissibility, immune evasion, and, in some cases, increased disease severity. Key drivers of VOC emergence include immune pressure, host adaptation, prolonged infections in immunocompromised individuals, and global connectivity. Understanding these evolutionary mechanisms is crucial for refining mitigation strategies and guiding next-generation vaccine development. Mutations in the spike (S) protein, particularly within the RBD, such as E484K and N501Y, have been associated with reduced neutralizing antibody efficacy, facilitating immune escape and contributing to reinfections and breakthrough cases in vaccinated individuals [40,41]. Similarly, host adaptation plays a critical role, with mutations like D614G, observed early in the pandemic, improving spike protein stability and increasing viral infectivity [42]. Prolonged infections in immunocompromised individuals provide an ideal environment for the virus to accumulate mutations under selective pressure, leading to the emergence of highly divergent variants, such as those observed in the Omicron lineage. Moreover, global connectivity, facilitated by high rates of international travel and large population densities, amplifies the rapid dissemination of new variants, enabling them to dominate in different geographic regions [43].

The impacts of these evolutionary dynamics are evident in the characteristics and spread of major VOCs. Alpha (B.1.1.7), first detected in the UK in September 2020, was characterized by increased transmissibility due to key mutations like N501Y and P681H, which enhanced spike protein cleavage and binding to the ACE2 receptor. This variant spread rapidly across Europe and North America, leading to significant surges in cases and hospitalizations. Beta (B.1.351), identified in South Africa in late 2020, exemplified the virus’s ability to evade immune responses, with mutations such as E484K and K417N significantly reducing neutralization by convalescent plasma and vaccine-induced antibodies. This immune evasion necessitated the development of booster doses and updated vaccine formulations. Delta (B.1.617.2), which emerged in India, combined high transmissibility with increased disease severity. Its key mutations, including L452R and P681R, enhanced ACE2 receptor binding and cell entry, resulting in devastating waves of infections and overwhelming healthcare systems worldwide. Omicron (B.1.1.529) marked a new chapter in the pandemic, with over 30 mutations in the spike protein alone. These extensive genetic changes allowed Omicron to evade immunity from vaccines and prior infections, causing widespread reinfections and breakthrough cases. Despite its relatively milder clinical outcomes, Omicron’s high transmissibility led to overwhelming case numbers and significant strain on global healthcare systems [44,45,46]. Some key characteristic features of these SARS-CoV-2 VOCs, including their major mutations, virulence traits, vaccine efficacy, and global impact, are summarized in Table 1.

The public health implications of VOCs extend beyond their virological properties, significantly influencing pandemic management strategies. Variants like Delta and Omicron, with higher transmissibility and immune evasion, have driven successive infection waves, often overwhelming healthcare systems. Their elevated reproduction numbers (R0) necessitated stringent public health measures, including travel restrictions, lockdowns, mask mandates, and mass vaccination campaigns. However, these interventions have underscored global inequities, with low- and middle-income countries facing limited access to vaccines, diagnostics, and therapeutics [56,57]. Immune evasion by VOCs, particularly Beta and Omicron, has reduced vaccine effectiveness, prompting widespread booster administration and the development of bivalent vaccines. Despite these efforts, reinfections and breakthrough cases remain common, highlighting the need for continuous vaccine research and next-generation immunogens to address emerging SARS-CoV-2 lineages [58,59].

The clinical severity of VOCs varies, influencing healthcare burdens differently. Alpha and Delta variants were associated with higher hospitalization and mortality rates, exacerbating pressure on healthcare systems. In contrast, Omicron, despite its milder clinical presentation, caused immense strain due to the sheer volume of cases, particularly in regions with low vaccination rates or fragile healthcare infrastructures. This surge in cases, compounded by limited access to therapeutics in many areas, has underscored the importance of equitable resource allocation and global solidarity in addressing the pandemic [60,61]. Beyond direct health impacts, VOCs have prolonged economic and social disruptions. Recurring waves driven by these variants have resulted in intermittent lockdowns, school closures, and disruptions in global supply chains, compounding the socio-economic toll of the pandemic. Small businesses, healthcare systems, and education sectors have borne the brunt of these disruptions, highlighting the need for comprehensive strategies that balance public health priorities with economic stability [62,63].

The emergence of VOCs has also emphasized the importance of genomic surveillance as a cornerstone of pandemic response. Robust surveillance systems enable the early detection and characterization of emerging variants, providing critical data to inform public health strategies and vaccine updates [64]. Investment in genomic sequencing capacity and data-sharing mechanisms is essential for ensuring timely responses to new threats. Vaccine development and manufacturing have similarly taken center stage, with mRNA vaccine platforms offering the flexibility to adapt formulations quickly in response to evolving variants. Equitable distribution of vaccines and therapeutics remains a pressing concern, as global disparities in access continue to hinder efforts to control the pandemic. Collaborative initiatives such as COVAX aim to address these disparities, but challenges persist in achieving widespread coverage in low-resource settings [65,66,67].

Antiviral therapies represent another critical tool for mitigating the impact of VOCs. Agents like paxlovid and molnupiravir have demonstrated efficacy against severe disease and hospitalization, providing a complementary approach to vaccination [68,69]. However, the potential for antiviral resistance highlights the need for prudent use and continued research into novel therapeutic agents. Public health interventions must remain flexible and data-driven, balancing the need for stringent measures with consideration for economic and social well-being. As SARS-CoV-2 continues to evolve, the global response must integrate scientific innovation with coordinated public health measures to anticipate and address emerging threats effectively.

In conclusion, the emergence and spread of SARS-CoV-2 VOCs underscore the dynamic interplay between viral evolution and public health measures. These variants exemplify the virus’s adaptability and its potential to exploit vulnerabilities in human populations, driving successive waves of infections and prolonging the pandemic. Addressing the challenges posed by VOCs requires a coordinated global strategy that integrates genomic surveillance, vaccine innovation, antiviral development, and equitable resource allocation. As the pandemic persists, maintaining a proactive stance and leveraging both scientific advancements and robust public health frameworks will be critical in mitigating the impact of SARS-CoV-2 and preparing for future outbreaks of this scale. The lessons learned from managing VOCs must guide efforts to strengthen global health systems, ensuring resilience against emerging infectious diseases in the years to come.

## 3. Major SARS-CoV-2 Variants of Concern (VOCs) Emerged During the Pandemic Era

### 3.1. Alpha Variant (B.1.1.7)

The Alpha variant (B.1.1.7) emerged as the first significant SARS-CoV-2 VOCs to reshape the global trajectory of the pandemic. Detected in the United Kingdom in September 2020, it exhibited a notable increase in transmissibility compared to earlier strains, attributed to specific mutations in the spike protein [70]. The N501Y mutation enhanced the virus’s ability to bind the ACE2 receptor, a crucial gateway for viral entry into human cells, thereby increasing the infection rate. Another critical mutation, P681H, improved cleavage efficiency at the furin cleavage site of the spike protein, facilitating quicker and more effective viral entry. The deletion of amino acids 69–70 in the spike protein not only increased infectivity but also interfered with certain PCR diagnostic assays, which initially led to underreporting in some regions [71,72,73]. The transmissibility of Alpha was estimated to be 50–70% higher than the original Wuhan strain, with an R0 of approximately 4–5, which allowed it to spread rapidly within and beyond the UK [74,75,76]. Alpha’s global impact was profound. By early 2021, it became the dominant strain in many countries, including the United States and much of Europe. The variant drove a severe winter surge in the UK, necessitating nationwide lockdowns to curb hospitalization and mortality. During the winter of 2020–2021, daily case numbers in the UK exceeded 60,000 at their peak, and hospitals faced unprecedented pressure, with intensive care units reaching near-capacity levels. Vaccination efforts were accelerated to combat the variant’s rapid spread, with the rollout prioritizing high-risk groups [74,77]. In the United States, Alpha accounted for more than half of COVID-19 cases by April 2021, exacerbating regional surges in areas with lower vaccination rates. In Michigan, for example, the Alpha variant drove a wave of cases that overwhelmed healthcare systems, underscoring the need for consistent vaccine coverage across all states [78].

### 3.2. Beta Variant (B.1.351)

The Beta variant (B.1.351), first detected in South Africa in late 2020, exhibited significant immune evasion, primarily due to mutations in the RBD of the spike protein, including E484K and K417N, which reduced the efficacy of nAbs. Additionally, the N501Y mutation, shared with the Alpha variant, enhanced transmissibility, although Beta had a lower reproduction number (R0 ≈ 3–4), leading to slower spread. Its ability to evade immunity, particularly from natural infection and adenoviral vector-based vaccines, raised global concerns about the effectiveness of first-generation vaccines [70]. Beta’s impact was most pronounced during South Africa’s second wave of infections in late 2020 and early 2021. The surge overwhelmed hospitals, with daily cases surpassing previous records, and prompted the government to impose stricter restrictions. The emergence of Beta coincided with clinical trial data showing that the AstraZeneca vaccine offered limited protection against mild to moderate illness caused by this variant. Consequently, South Africa shifted its vaccination strategy, opting for the single-dose Johnson & Johnson vaccine, which demonstrated greater efficacy against Beta. Internationally, the variant’s spread to countries such as France and South Korea heightened concerns about vaccine efficacy and reinforced the need for booster doses. For instance, France reported clusters of Beta infections in early 2021, leading to increased genomic surveillance and tighter travel restrictions from regions where Beta was prevalent [79,80,81].

### 3.3. Gamma Variant (P.1)

The Gamma variant (P.1), first identified in Manaus, Brazil, in November 2020, posed a unique threat due to its ability to cause reinfections in populations previously thought to have high levels of natural immunity. Manaus had experienced a severe COVID-19 outbreak in early 2020, with seroprevalence studies suggesting that over 75% of the population had been exposed to the virus. Despite this, Gamma drove a catastrophic second wave in early 2021, overwhelming the city’s healthcare system and resulting in a dramatic spike in mortality. This resurgence highlighted the variant’s immune evasion capabilities, attributed to mutations such as E484K and K417T in the RBD, which reduced the efficacy of antibodies generated by prior infections. The N501Y mutation also contributed to increased transmissibility, with an R0 of approximately 4 [70]. Gamma’s global impact, while less widespread than Alpha or Delta, was significant in regions with high seroprevalence from earlier waves. In Brazil, the variant’s emergence led to prolonged national outbreaks, with hospitals across multiple states reporting critical shortages of oxygen and intensive care beds. Outside Brazil, the introduction of Gamma into Japan via travelers highlighted the importance of border controls and quarantine measures. Japan implemented stricter screening protocols and genomic surveillance to prevent the widespread transmission of Gamma within its borders, a strategy that successfully limited its domestic impact. Nevertheless, Gamma underscored the risks posed by variants capable of evading natural immunity and reinforced the global focus on vaccine adaptations [82,83,84,85].

### 3.4. Delta Variant (B.1.617.2)

The Delta variant (B.1.617.2), first detected in India in October 2020, was a defining force in the pandemic due to its unparalleled transmissibility and association with more severe disease outcomes. Key mutations, such as L452R and P681R, enhanced Delta’s ability to bind the ACE2 receptor and increased the efficiency of viral entry into human cells. Delta’s transmissibility was approximately 60% higher than Alpha, with an R0 estimated at 5–8. This variant also exhibited increased viral load in infected individuals and a longer duration of infectiousness, further amplifying its spread. Delta’s impact was devastating in India during the second wave of infections in early 2021. Daily case counts exceeded 400,000 at the peak, and hospitals across the country were overwhelmed. Severe shortages of oxygen, antiviral medications, and hospital beds led to widespread suffering and high mortality rates. The Delta-driven wave prompted an international outpouring of aid, with countries sending medical supplies to alleviate the crisis. Globally, Delta quickly became the dominant strain, driving surges in cases and hospitalizations even in countries with relatively high vaccination rates [86,87,88,89,90].

### 3.5. Omicron Variant (B.1.1.529)

The Omicron variant (B.1.1.529), first identified in South Africa and Botswana in November 2021, represented a paradigm shift in the pandemic due to its extensive immune evasion and extraordinary transmissibility. With over 50 mutations, including more than 30 in the spike protein, Omicron demonstrated remarkable genetic divergence from earlier variants. Mutations such as E484A, G446S, and multiple deletions in the spike protein allowed Omicron to evade nAbs generated by prior infections and vaccinations. While its intrinsic virulence was lower than that of Delta, Omicron’s transmissibility, with an R0 estimated at 10–15, resulted in massive surges in cases worldwide [44]. In South Africa, Omicron drove a sharp increase in cases in late 2021, although hospitalization rates were lower compared to previous waves. The variant’s emergence highlighted the importance of booster doses, as studies indicated that two-dose vaccine regimens provided limited protection against infection but were more effective at preventing severe disease when supplemented with a booster [91,92]. Globally, Omicron caused record-breaking daily case counts in early 2022. In the United States, Omicron infections surged to over one million cases per day, straining healthcare systems despite high vaccination rates. The emergence of sub-lineages such as BA.1, BA.2, and XBB further underscored Omicron’s adaptability and the need for continuous genomic surveillance and vaccine updates [93,94]. The global impact of these variants collectively reshaped public health strategies and highlighted the importance of adaptability in pandemic response. Alpha and Delta underscored the urgency of rapid vaccine rollouts to mitigate severe disease and high transmissibility. Beta and Gamma emphasized the need for vaccine adaptations to address immune evasion, while Omicron reaffirmed the critical importance of booster doses and equitable vaccine distribution. Each variant’s unique characteristics and global repercussions provide invaluable lessons for managing future pandemics and improving global health preparedness [44].

## 4. Newly Emerged SARS-CoV-2 Variants in the Post-Pandemic Era

The post-pandemic era (2023–2024) of COVID-19 witnessed the emergence of new SARS-CoV-2 variants and subvariants, indicating a period of ongoing viral evolution. Various genetic mutations have driven the development of these variants, which affect their transmissibility, immune evasion, and potential clinical impact.

### 4.1. Spring–Summer 2022: Emergence of BA.4 and BA.5

By late March 2022, the BA.4 and BA.5 subvariants of SARS-CoV-2 were identified in South Africa. These variants emerged through recombination events, resulting in complex ancestry, making it unclear whether they are direct descendants or sister lineages of BA.2 [95]. Although BA.4 and BA.5 share identical S proteins, they differ from BA.2 by harboring a deletion (HV69-70del) in the N-terminal domain (NTD) and three amino acid substitutions (L452R, F486V, and R493Q [a revertant]) in the RBD. Due to their higher effective reproduction number (Re), approximately 1.3 times greater than that of BA.2 in South Africa, both variants rapidly spread worldwide. However, BA.5 eventually became the dominant strain, likely due to its slightly higher transmission advantage [96].

### 4.2. Summer 2022: Emergence of BA.2.75

In May 2022, the BA.2.75 and BJ.1 sub-lineages, both second-generation derivatives of BA.2 with an increased number of mutations, were identified in India. Compared to BA.2, BA.2.75 harbors nine additional amino acid substitutions in the S protein—five in the NTD (K147E, W152R, F157L, I210V, and G257S) and four in the RBD (D339H, G446S, N460K, and R493Q) [97]. When BA.2 was prevalent in India, BA.2.75 exhibited a higher transmission advantage, with a Re approximately 1.35 times that of BA.2. However, BA.2.75 failed to achieve significant global dominance in regions where BA.5 was already established, likely due to BA.5’s superior transmissibility. Studies using human sera and pseudoviruses expressing BA.2-based S proteins have shown that the F486V substitution in BA.5 enhances immune evasion by reducing susceptibility to nAbs. However, this mutation simultaneously decreases the affinity of the spike protein for the ACE2 receptor. To counteract this loss of binding efficiency, BA.5 acquired the L452R mutation—previously observed in the Delta variant—which enhances ACE2-binding affinity, contributing to its higher transmissibility and competitive advantage [98].

### 4.3. Autumn–Winter 2022: Emergence of BQ.1.1 and XBB Lineages

Following the global spread of BA.5, several SARS-CoV-2 variants with comparable Re values emerged in different regions, leading to a complex epidemiological scenario in late 2022, often termed the “variant soup” [99]. During this period, multiple variants coexisted without a single dominant strain. Among these, BQ.1.1 and XBB were particularly notable. BQ.1.1 (BA.5.3.1.1), a descendant of BA.5, which was first detected in Africa and Europe, acquired key S protein mutations—K444T, N460K, and R346T—sequentially, contributing to increased immune evasion and transmissibility [100]. In contrast, XBB arose from a recombination event between two second-generation BA.2 sub-lineages—BA.2.75 (specifically BM.1.1.1; BA.2.75.3.1.1.1) and BJ.1. This recombination resulted in XBB carrying 14 additional mutations compared to BA.2, including nine in the RBD and five in the NTD. Both BQ.1.1 and XBB exhibited higher transmissibility, with Re values approximately 1.25 times greater than BA.5, which led to a shift in dominance from BA.5 to these emerging variants in late 2022 [101]. The F486V mutation, as observed in BA.5, plays a crucial role in antibody evasion in the BQ.1 lineage. However, this mutation reduces ACE2 binding affinity. To compensate for this, BQ.1.1 acquired the N460K mutation, originally identified in BA.2.75. Structural studies using X-ray crystallography revealed that N460K in the S protein forms a glycan-mediated interaction network with the N90-linked glycan on ACE2, involving contacts with N405 of the S protein and R559 of ACE2. This interaction network helped to stabilize the ACE2–RBD binding, enhancing viral attachment [100]. Thus, BQ.1.1 represents a convergent evolutionary variant, where the combined effects of F486V and N460K, inherited from BA.5 and BA.2.75, respectively, improved both ACE2 binding affinity and immune evasion, ultimately enhancing viral fitness. In XBB.1, the F486 residue was replaced with serine (F486S), a hydrophilic substitution that significantly reduced ACE2 binding affinity compared to its ancestral form, BA.2.75. However, this mutation markedly increased neutralizing antibody evasion, contributing to its enhanced immune escape properties. Consequently, XBB.1 evolved as a convergent variant, acquiring a combination of F486S and N460K, which collectively improved viral fitness by optimizing immune evasion despite reduced ACE2 affinity [101,102].

### 4.4. Winter 2022–Spring 2023: Emergence of XBB.1.5

XBB.1.5, a descendant of the XBB lineage, acquired the F486P substitution, which significantly enhanced its transmissibility and immune evasion. This variant rapidly spread across the United States from late November 2022. The F486P mutation conferred a 1.2-fold higher Re compared to XBB.1 and BQ.1.1, contributing to its rapid dominance [102].

### 4.5. Spring–Summer 2023: Emergence of EG.5.1

EG.5.1 (XBB.1.9.2.5.1), an XBB.1.5 descendant, acquired additional spike protein mutations, Q52H and F456L, which further enhanced viral fitness. EG.5.1 exhibited a 1.2-fold higher Re than XBB.1.5, facilitating its widespread transmission across East and Southeast Asia and North America [103].

### 4.6. Summer 2023–Winter 2024: Emergence of “FLip” Variants

Subsequent evolution of XBB sub-lineages, such as HK.3, introduced additional mutations, including L455F, alongside F486P and F456L (as seen in EG.5.1). These variants were termed “FLip” variants due to the amino acid switch from LF to FL at positions 455 and 456 [104]. Among these, HK.3 (EG.5.1.1.3; XBB.1.9.2.5.1.1.3), a descendant of EG.5.1 first identified in China, demonstrated the highest Re value, making it a highly competitive variant within the XBB lineage [105].

### 4.7. Summer 2023–2024: Emergence of JN.1 and Other BA.2.86 Lineages

In summer 2023, BA.2.86, a phylogenetically distinct lineage separates from XBB, emerged with approximately 30 mutations in the S protein. Unlike the rapidly spreading XBB sub-lineages, BA.2.86 exhibited a gradual global dissemination [106]. Despite its distinct evolutionary trajectory, BA.2.86 had Re comparable to HK.3, which allowed both variants to co-circulate in certain regions. A notable descendant of BA.2.86, JN.1, acquired the L455S mutation, which significantly enhanced its immune evasion capacity. By the end of 2023, JN.1 outcompeted and nearly replaced the XBB lineages in the human population, marking a major shift in SARS-CoV-2 variant dominance [107]. A study by Qingwen et al. evaluated the immune evasion properties of EG.5, EG.5.1, BA.2.86, and JN.1 using human sera, murine sera, and a panel of 41 mAbs targeting eight distinct RBD epitopes. The study found that JN.1 exhibited the highest degree of immune evasion, surpassing BA.2.86. Notably, BA.2.86 demonstrated lower neutralization resistance than EG.5 and EG.5.1, raising concerns about the evolutionary advantage conferred by JN.1’s additional mutations [108].

### 4.8. August–October 2024: Emergence of XEC and KP.3.1.1

As of October 2024, KP.3.1.1 (JN.1.11.1.3.1.1) has become the dominant SARS-CoV-2 variant worldwide, surpassing other JN.1-derived sub-lineages such as KP.2 (JN.1.11.1.2) and KP.3 (JN.1.11.1.3). KP.3.1.1 acquired the S:31del mutation, along with additional spike protein substitutions (S:R346T, S:F456L, and S:Q493E), enhancing its competitive advantage over previous JN.1 sub-lineages. On August 7, 2024, XEC, a recombinant lineage of KS.1.1 (JN.13.1.1.1) and KP.3.3 (JN.1.11.1.3.3), was first detected in Germany. This recombination event introduced two notable spike protein mutations (S:T22N and S:F59S), with a genomic breakpoint occurring at positions 21,738–22,599. Neutralization studies revealed that XEC had a 50% neutralization titer (NT50) approximately 1.3 times lower than KP.3.1.1, indicating a higher level of immune evasion. Additionally, both S:T22N and S:F59S mutations conferred a 1.5- to 1.6-fold increase in resistance to KP.3.3 breakthrough infection (BTI) sera. Functionally, XEC exhibited enhanced immune escape and pseudovirus infectivity compared to KP.3. Moreover, XEC demonstrated greater immune resistance to KP.3.3 BTI sera than KP.3.1.1, further highlighting its potential to influence SARS-CoV-2 evolution [109,110].

## 5. SARS-CoV-2 Vaccines: Platforms, Efficacy, and Performance Against Variants

### 5.1. SARS-CoV-2 Vaccine Platforms: First-Generation and Beyond

The COVID-19 pandemic has accelerated vaccine development through innovative platforms. Understanding how these vaccines elicit immune responses and confer protection is crucial for optimizing existing vaccines and designing new ones against emerging infections. This section explores the mechanisms of action of various COVID-19 vaccine platforms—including mRNA, viral vector, inactivated virus vaccines, and protein subunit vaccines—with a focus on their ability to stimulate immune responses and provide protection.

Moderna and Pfizer/BioNTech were the first to receive FDA Emergency Use Authorization (EUA) and EMA conditional approval for their mRNA-based COVID-19 vaccines. Moderna developed mRNA-1273, a two-dose intramuscular vaccine encoding the full-length SARS-CoV-2 S protein, which demonstrated strong efficacy in clinical trials. Pfizer/BioNTech created multiple lipid nanoparticle (LNP)-encapsulated mRNA vaccine candidates (BNT162a1, b1, b2, c2), ultimately advancing BNT162b2, which encodes the SARS-CoV-2 RBD, due to its favorable safety profile and robust neutralizing antibody response. Whereas BNT162b1, encoding a trimerized, secreted form of the S protein RBD, elicited strong CD4^+^ and CD8^+^ T-cell responses and demonstrated safety and immunogenicity in a phase 1 trial [111,112,113]. Other companies, such as CureVac, have also developed mRNA vaccines, including CVnCoV, which encodes the S protein and is administered in two doses using LNP encapsulation [114]. The Moderna and Pfizer mRNA vaccines share a similar mechanism of action. Encapsulated in LNPs for efficient delivery, they contain nucleoside-modified mRNA encoding the SARS-CoV-2 spike glycoprotein with a transmembrane anchor. These vaccines elicit both T-cell and B-cell responses, generating antibodies against the RBD and full-length S2-P. Their efficacy is enhanced by LNPs, which facilitate cellular uptake, and modified nucleotides that prevent premature activation of interferon-associated genes [115]. Additionally, they stimulate germinal center B cells, induce antigen-specific T-follicular helper cells, and promote prolonged antigen expression, ensuring a robust and durable immune response [116]. A two-dose regimen of BNT162b2 (30 µg per dose administered 21 days apart) demonstrated a favorable safety profile and 95% efficacy in preventing symptomatic COVID-19, as reported in a large placebo-controlled Phase III clinical trial involving over 37,000 participants [117]. Subsequent studies have shown that the vaccine induces a more robust immunogenic response in adolescents and children compared to young adults, with higher neutralizing antibody titers and T cell responses observed in these age groups [118]. The majority of vaccine recipients experienced transient mild-to-moderate local and systemic side effects—such as injection site pain, fatigue, headache, and fever—more frequently following the second dose, consistent with the development of a strong adaptive immune response [112]. While generally well tolerated, rare cases of anaphylaxis have been reported, predominantly in individuals with a known history of severe allergies [119,120]. Additionally, few isolated cases of myocarditis have been reported within two weeks of vaccination, although a definitive causal relationship has not been fully established [121].

Viral vector vaccines, such as Oxford–AstraZeneca’s ChAdOx1 nCoV-19 (AZD1222), have demonstrated efficacy ranging from 66.7% to 70.4% against symptomatic ancestral SARS-CoV-2 infection, based on interim analyses from ongoing multinational Phase III randomized controlled trials [122,123]. Marketed as Vaxzevria, this vaccine utilizes a chimpanzee adenovirus vector carrying the SARS-CoV-2 spike protein gene with a tissue plasminogen activator (tPA) leader sequence. Other adenovirus-based vaccines include Johnson & Johnson’s Ad26.COV2.S, which employs a modified adenovirus 26 vector, and Sputnik V, a dual-vector vaccine using Ad5 and rAd26 to encode the spike protein [124]. These vaccines deliver viral DNA into host cells via non-replicating adenoviral vectors, leading to the expression of the SARS-CoV-2 spike protein, which in turn stimulates both humoral and cellular immune responses, including the production of nAbs. As these vectors are replication-incompetent, they cannot cause SARS-CoV-2 infection, yet they are capable of inducing protective immunity. While generally well tolerated, with commonly reported mild-to-moderate side effects such as fatigue, headache, and low-grade fever, rare but serious adverse events have also been documented. Among these, thrombotic events associated with thrombocytopenia have raised clinical concern. This condition, known as vaccine-induced immune thrombotic thrombocytopenia (VITT) or thrombosis with thrombocytopenia syndrome (TTS), has been observed predominantly in individuals under 60 years of age—particularly women—typically occurring within 5 to 28 days post-vaccination [125,126,127,128]. These cases are often associated with high levels of anti-platelet factor 4 (PF4) antibodies, resembling the immunopathogenesis of autoimmune heparin-induced thrombocytopenia (HIT) [129,130,131]. Thromboses have been reported at unusual sites, including the cerebral venous sinuses, splanchnic veins, and pulmonary arteries [125,132]. Given the potential risks, it is essential that recipients of the ChAdOx1 nCoV-19 vaccine are counseled about the signs and symptoms of thrombotic thrombocytopenia and are encouraged to seek immediate medical attention should such symptoms occur.

Inactivated COVID-19 vaccines, including CoronaVac (Sinovac), Sinopharm, BBIBP-CorV, and Covaxin, are developed by chemically or physically inactivating live SARS-CoV-2 using heat, ultraviolet light, or chemical agents like β-propiolactone or formaldehyde. While these vaccines cannot replicate or cause infection, they effectively stimulate the immune system upon exposure to the virus. They are particularly suitable for immunocompromised individuals due to their safety profile. However, they generally elicit a weaker immune response compared to live vaccines and often require multiple booster doses. Additionally, their production is time-intensive, as the virus must be cultured before inactivation [133,134]. Phase III clinical trials of CoronaVac have been conducted in various countries, demonstrating variable efficacy outcomes—50.7% in Brazil, 65.3% in Indonesia, and 83.5% in Turkey [51,135,136]. The most commonly reported side effects included pain at the injection site and mild systemic reactions. Compared to viral vector or nucleic acid-based vaccines, the incidence of fever following CoronaVac immunization was notably lower [137], and the vaccine was better tolerated among individuals aged over 60 years [138]. However, a decline in nAb titers with increasing age has been observed, suggesting that older adults may benefit from higher doses or additional booster shots to achieve sufficient immune protection [139]. CoronaVac is considered safe and poses no risk of causing infection, even in immunocompromised individuals, as it contains inactivated viruses that cannot replicate in the human body. Nevertheless, inactivated vaccines generally induce weaker immunogenicity compared to live-attenuated vaccines, as they predominantly stimulate humoral immunity with limited T cell-mediated responses [140,141]. Consequently, multiple doses are often required to elicit a robust and sustained immune response. Although no evidence of antibody-dependent enhancement (ADE) has been observed in human trials of CoronaVac, theoretical concerns remain. Inactivated vaccines have the potential to induce non-neutralizing antibodies, which could contribute to immunopathology such as vaccine-associated enhanced respiratory disease (VAERD), as previously observed with inactivated vaccines against respiratory syncytial virus (RSV) and measles [142,143,144,145].

Novavax’s NVX-CoV2373 is a recombinant protein vaccine designed to protect against SARS-CoV-2 and its variants. It uses a highly purified recombinant spike (rS) protein nanoparticle, produced through a baculovirus-insect cell system and derived from the original Wuhan-Hu-1 strain. The protein includes modifications, such as two proline substitutions in the S2 domain and amino acid alterations in the S1/S2 cleavage site, to stabilize the prefusion conformation and improve protease resistance [146]. The vaccine, formulated with the Matrix-M™ adjuvant, enhances immune responses, reduces antigen requirements, and maintains a strong safety profile, as demonstrated in trials with over 37,000 participants. Antibodies generated by the vaccine block the SARS-CoV-2 spike protein from binding to the human ACE2 receptor [147].

### 5.2. Vaccine Efficacy Against SARS-CoV-2 VOCs and Emerging Sub-Variants

The concept of VOCs—viral mutations that raise concerns due to their potential impact on transmissibility, disease severity, immune escape, and vaccine effectiveness—is based on the historical lessons learned from virology. Research on influenza has demonstrated that both antigenic drift and antigenic shift in viral pathogens can significantly affect vaccine effectiveness. Antigenic drift refers to the gradual and continuous accumulation of mutations in viral genes encoding surface proteins leading to changes in antigenicity over time and across seasons. In contrast, antigenic shift involves an abrupt and substantial reassortment of genetic material, often resulting from co-infections leading to the emergence of a novel viral subtype with significantly altered antigenic properties. This process can precipitate pandemics due to the population’s limited pre-existing immunity [148]. Insights gained from both seasonal and pandemic influenza, such as the 2009 H1N1 pandemic, have influenced global strategies for monitoring and responding to SARS-CoV-2 mutations [149,150,151]. Studies on influenza viruses have long established that antigenic drift is a recurring phenomenon, necessitating the continuous update of seasonal influenza vaccines to maintain efficacy. The influenza virus frequently modifies its surface antigens, particularly HA and NA, requiring the annual reformulation of flu vaccines to match circulating strains. Similar considerations apply to SARS-CoV-2, where evolving variants necessitate updated vaccine formulations to sustain protective immunity [152,153,154]. Similarly, the vaccines developed in 2020 against SARS-CoV-2 were designed to elicit an immune response against the spike protein of the ancestral Wuhan-Hu-1 strain. These vaccines were highly effective at the time, particularly in preventing severe disease, hospitalization, and death. However, as SARS-CoV-2 evolved, accumulating mutations in the spike protein—especially in key antigenic regions—some variants exhibited partial immune escape, reducing the neutralizing activity of vaccine-induced antibodies [15,155,156] (Table 2).

The emergence of early variants, such as Alpha (B.1.1.7) and Beta (B.1.351), marked the beginning of significant viral evolution [158]. The Alpha variant, first detected in late 2020, was associated with increased transmissibility due to mutations that enhanced viral binding to the ACE2 receptor [159]. The Beta variant, which contained several spike protein mutations, exhibited a higher degree of immune evasion, particularly affecting neutralization by vaccine-induced antibodies. This effect was more pronounced against vaccines like Pfizer-BioNTech (BNT162b2) and AstraZeneca (ChAdOx1 nCoV-19), leading to reduced efficacy against mild to moderate infection, though protection against severe disease remained relatively strong [160]. The Delta variant (B.1.617.2), which became globally dominant by mid-2021, was characterized by an even greater increase in transmissibility and virulence, particularly among unvaccinated individuals [86]. Mutations in the spike protein not only enhanced viral replication and infectivity but also contributed to immune evasion, leading to reduced vaccine effectiveness against symptomatic infection. However, vaccines continued to provide substantial protection against severe disease and hospitalization. A UK study among individuals aged over 70 found that two doses of the Pfizer-BioNTech vaccine (BNT162b2) were approximately 88% effective against symptomatic infection caused by the Delta variant, compared to over 95% effectiveness against the original Wuhan strain [47].

The most heavily mutated SARS-CoV-2 variant identified at that time was Omicron (B.1.1.529), which emerged in late 2021. Omicron harbored more than 30 mutations in the spike protein, approximately two-thirds of which were located in the RBD [161]. The rapid global spread of Omicron was largely driven by its ability to evade immunity induced by both vaccination and prior infections. By early 2022, Omicron and its sub-lineages (e.g., BA.1, BA.2, BA.5) had become dominant worldwide, fueling large-scale infections even among vaccinated individuals [162,163,164,165]. Despite Omicron’s extensive immune escape, vaccination—particularly with booster doses—continued to provide substantial protection against severe disease, hospitalization, and death [166]. A study by Andrews et al. reported that while vaccine effectiveness against symptomatic infection was reduced to approximately 30% for Omicron, protection against severe outcomes remained high, ranging from 80% to 90%, particularly among those who received booster doses [46]. Similarly, a nationwide vaccination analysis in Israel found that booster doses increased protection by more than 80% against severe disease during the Omicron wave [167]. In response to Omicron’s immune evasion, updated vaccine candidates were developed. The most notable among them were the bivalent vaccines, which targeted both the original Wuhan-Hu-1 strain and Omicron subvariants. These updated vaccines were introduced in late 2022 and demonstrated greater effectiveness than the original monovalent vaccines, particularly in preventing severe disease caused by Omicron variants, as shown in real-world clinical data [58,168]. Global data underscored the critical role of booster doses, especially for vulnerable populations such as the elderly. Studies from Brazil and South Africa found that booster doses during Omicron surges reduced the risk of hospitalization by approximately 85% [169]. In the United States, older adults were more prone to waning immunity, necessitating additional booster doses to maintain adequate protection. This pattern parallels the need for periodic updates in influenza vaccines and highlights the importance of tailored vaccination strategies for older and immunocompromised individuals [170].

### 5.3. Booster Doses and the Dynamics of Adaptive Immunity

With the progression of the COVID-19 pandemic, it became evident that the protective immunity conferred by the primary two-dose regimen of most vaccines waned over time. This pattern aligns with other viral vaccines, such as those for hepatitis B and influenza, where booster doses are integral to maintaining long-term immunity [171,172,173]. The emergence of SARS-CoV-2 variants, including Delta and Omicron, further highlighted the necessity of booster doses to restore and enhance immune protection against infection and severe disease [174]. Booster vaccinations function by re-stimulating the immune system, elevating both antibody titers and memory B and T cell responses. Early studies demonstrated a significant increase in neutralizing antibody levels following booster administration, particularly against the Delta variant [175]. Large-scale observational studies, such as those conducted in Israel in mid-2021, reported that individuals receiving a third dose of the Pfizer-BioNTech vaccine exhibited up to 90% protection against severe disease during the Delta surge, largely attributable to the rise in nAbs [176,177]. The high degree of spike protein mutations in the Omicron variant further underscored the importance of boosters. Initial studies indicated that two doses of mRNA vaccines provided minimal protection against symptomatic Omicron infection; however, booster doses reinstated strong protection against severe disease [178]. Waning immunity over time contributed to increased susceptibility to breakthrough infections, as evidenced by studies from the US and Europe showing a decline in neutralizing antibody titers within six months of the second vaccine dose [179,180]. Booster doses, particularly those reformulated to target Omicron subvariants, significantly enhanced both humoral and cellular immune responses.

While nAbs play a critical role in preventing infection, long-term immunity against severe disease is predominantly mediated by adaptive immune mechanisms, particularly T cells and memory B cells. Studies on other coronaviruses, such as SARS-CoV and MERS-CoV, have demonstrated that T-cell immunity can persist for years, even in the absence of detectable antibodies. This holds true for SARS-CoV-2, where booster doses not only augment neutralizing antibody production but also activate long-lived memory B and T cell responses [181,182]. Notably, while Omicron and its subvariants exhibit substantial immune evasion from nAbs, T-cell responses remain largely preserved, continuing to provide robust protection against severe disease. Multiple studies have confirmed that vaccinated individuals with strong T-cell responses were less likely to develop severe illness, even when infected with highly mutated variants such as BA.2 and BA.5 [183,184]. Despite the rapid increase in antibody levels following booster administration, the duration of this protection remains an area of active investigation. A study from Brazil demonstrated that although antibody titers declined within six months post-booster, protection against severe disease persisted for up to nine months, even amid the circulation of new Omicron subvariants [185]. Moving forward, periodic booster doses, potentially updated to match emerging variants, will likely be necessary to sustain immunity at the population level.

### 5.4. Determinants of Vaccine Performance: Host, Viral, and Environmental Factors

Vaccine efficacy is influenced by several host-related factors, including age, sex, comorbidities, prior infection history, and immune system variability. Research on influenza and SARS-CoV-2 vaccines has demonstrated that immune responses to vaccination vary significantly across populations. Age is a well-established determinant, with older adults exhibiting reduced vaccine efficacy due to immunosenescence [186,187]. Studies on SARS-CoV-2 vaccines have shown that neutralizing antibody titers decline more rapidly in individuals over 65, leading to diminished protection against symptomatic infection and severe disease. However, booster doses restore immunity, and vaccines remain highly effective in preventing severe outcomes and mortality, even with the emergence of variants such as Omicron and its sub-lineages [188,189].

Comorbidities such as diabetes, hypertension, cardiovascular disease, and obesity also contribute to reduced vaccine efficacy. Individuals with these conditions not only face higher risks of severe COVID-19 outcomes but also exhibit weaker vaccine-induced immune responses [190]. For instance, obesity has been linked to lower neutralizing antibody titers following mRNA vaccination, potentially increasing the likelihood of breakthrough infections [191,192]. Similarly, immunocompromised individuals, including those undergoing chemotherapy or living with HIV, show suboptimal responses to SARS-CoV-2 vaccination [193].

Waning immunity is a well-documented phenomenon in vaccinology, observed in both influenza and SARS-CoV-2 vaccines. While these vaccines initially elicit robust antibody responses, antibody levels decline over time, reducing protection against infection. For SARS-CoV-2, vaccine efficacy against symptomatic disease drops significantly after six months, particularly in the absence of booster doses [194]. However, protection against severe disease remains more durable due to persistent memory T-cell and B-cell responses [195]. Booster doses effectively counteract waning immunity, as demonstrated in a UK study where individuals who received an mRNA booster exhibited a 39-fold increase in neutralizing antibody titers compared to those who received only the primary two dose series [196]. Hybrid immunity, derived from both vaccination and prior infection, provides superior and longer-lasting protection compared to vaccine-induced immunity alone. A large study from Qatar demonstrated that hybrid immunity significantly enhances protection against hospitalization and severe disease, even against emerging variants like BA.5 and XBB [197]. Studies on SARS and MERS suggest that prior infection can enhance vaccine-induced immune responses. A study by Le Bert et al. demonstrated that T cells from individuals who recovered from SARS-CoV-1 infection in 2003 exhibit strong cross-reactivity with SARS-CoV-2 proteins, including the nucleocapsid and non-structural proteins, indicating that conserved coronavirus regions can sustain long-term memory T cell responses [198]. Notably, these memory T cells remained functional for at least 17 years post-infection, suggesting that cellular immunity provides more durable protection than humoral responses. Cross-reactive T cell immunity plays a crucial role in hybrid immunity and provides key insights for the development of pan-coronavirus vaccines by highlighting how conserved T cell epitopes can elicit broad and long-lasting protection [198].

Genetic factors also play a crucial role in modulating vaccine responses. Variations in immune-related genes, such as HLA alleles and cytokine pathways, influence individual susceptibility to infection and vaccination outcomes. Recent studies have identified specific genetic polymorphisms associated with stronger or weaker antibody responses to mRNA vaccines. A deeper understanding of these genetic determinants might support future vaccination strategies, optimizing protection across diverse populations [199].

## 6. Recent Advancements in Vaccine Technology with Enhanced Breadth of Protection

### 6.1. Multivalent Vaccines: Targeting Multiple SARS-CoV-2 Variants

Multivalent vaccines have demonstrated efficacy in resisting and preventing both existing and emerging strains of COVID-19. A significant study examines the urgent need for multivalent vaccines and the shortcomings of monovalent vaccines, focusing on the interaction between vaccination and SARS-CoV-2 variants (Alpha, Delta, and Omicron) and its impact on the viral dynamics of infection in England. The study indicates that vaccination against the Alpha and Delta variants resulted in reduced durations of positivity, decreased viral loads, and milder disease severity compared to unvaccinated individuals. The emergence of the Omicron variant significantly reduced the benefits of vaccination in lowering viral load and the duration of infection in individuals. The research indicates that although vaccination reduced the viral dynamics of Alpha and Delta infections, this effect was diminished in the case of Omicron, highlighting the necessity for modified or multivalent vaccines [200].

Numerous studies have been published detailing the application of multivalent vaccines in clinical trials, along with strategies and designs for developing novel multivalent vaccines. A recent study examines a strategy for creating broadly protective multivalent COVID-19 vaccines utilizing an Adenoviral vector platform (Ad5/35). Multivalent vaccines are essential due to the emergence of vaccine-resistant SARS-CoV-2 variants, particularly the rapidly evolving SARS-CoV-2 Omicron sub-lineages, which have compromised the efficacy of current vaccines. In the study referenced, the authors developed bivalent and trivalent vaccines aimed at different Omicron variants utilizing an Ad5/35 vector platform. Research conducted on mice and macaques demonstrated that multivalent vaccines (Bivalent BA.5/BA.2.75, trivalent XBB/BN.1/BQ.1.1) displayed enhanced cross-neutralization activity relative to monovalent vaccines. The trivalent vaccines (XBB/BN.1/BQ.1.1 and XBB.1.5/BN.1/BQ.1.1) demonstrated enhanced neutralizing antibody responses against both current and emerging strains, including XBB.1.5, XBB.1.16, EG.5.1, FL.1.5.1, and BA.2.86. This evidence indicates that multivalent vaccine strategies may be effective in combating the ongoing mutation of the virus [201].

In addition to trivalent vaccines, research is underway to develop a tetravalent vaccine, which may provide protection against a larger array of future VOCs. A tetravalent vaccine under investigation by various groups is SCTVO1E, which incorporates spike proteins from the Alpha, Beta, Delta, and Omicron BA.1 variants and utilizes a squalene-based adjuvant. A report on the phase 3 clinical trial of SCTVO1E highlights its efficacy in preventing symptomatic SARS-CoV-2 infection, providing optimism amid the evolving landscape of SARS-CoV-2 subvariants. The study was a double-blind, randomized, placebo-controlled phase 3 clinical trial carried out in China during a time when Omicron variants were prevalent. In this trial, 9200 participants were randomized in a 1:1 ratio to receive either SCTVO1E or a placebo. SCTVO1E exhibited a vaccine efficacy of 69.4% seven days post-vaccination and 79.7% at 14 days post-vaccination. The vaccine demonstrates comparable efficacy in preventing both symptomatic SARS-CoV-2 infections and all infections, irrespective of symptom status, at 14 days following vaccination. The vaccine produced a 25-fold increase in neutralizing antibody response against Omicron BA.5, measured 28 days after vaccination. It was found to be generally safe and well-tolerated, with predominantly mild and short-lasting reactions, and no reported severe adverse events or deaths related to the vaccine [202]. A recent investigation examines the immune response and safety profile of SCTVO1E and the newly updated multivalent COVID-19 vaccine SCTV01E-1, in both unvaccinated individuals and those with prior vaccinations. The results demonstrate that both vaccines increased the levels of nAbs specific to Omicron BA.5. In unvaccinated individuals, nAbs titers increased by day 42, followed by stabilization after the administration of a booster (third) dose. Individuals previously vaccinated exhibited an increase in neutralizing antibody titers after receiving the second dose of SCTV01E or SCTV01E-1. The SCTV01E-1 and SCTV01E vaccines demonstrated a more robust neutralizing antibody response to Omicron BA.5 in both study cohorts, suggesting their viability as both a primary series and a booster option [203,204].

Recent advancements in the domain of multivalent vaccines have been noteworthy, featuring developments such as SCTV01C, SCTV01E-2, Bimervax, the Recombinant COVID-19 Trivalent (XBB + BA.5 + Delta) Protein Vaccine (Sf9 Cell), and the Novel Recombinant COVID-19 Bivalent, all contributing to significant progress in this area [202,204,205]. Multivalent COVID-19 vaccines provided extensive protection, while monovalent vaccines demonstrated effectiveness and immunogenicity against pandemic variants. Considering the complexities and the presence of various mutations and subvariants of SARS-CoV-2, it is essential to ensure a comprehensive and resilient COVID-19 vaccination strategy. A broader array of nAbs is generated when each variant presents novel neutralizing epitopes. Moreover, forthcoming strains may preserve mutant peptides recognized in various iterations, potentially augmenting cross-reactivity with emerging variants. The Alpha variant serves as a pertinent example, exhibiting a remarkable genetic similarity of 99.63% with the Omicron variant. The mutations identified in various strains, including Alpha, Beta, Delta, Gamma, and Omicron, contribute to an increased transmissibility. Notable mutations include T95I, G142D, K417N, T478K, N501Y, P681H, delta69/70, and delta145. To combat the rapid evolution of SARS-CoV-2, an effective approach involves the utilization of multivalent vaccines, which are meticulously chosen based on their antigenic properties [204,206,207]. Alongside the significant progress made in multivalent vaccines, it is crucial to expedite the development of next-generation vaccines to guarantee cross-protection against new and emerging variants.

### 6.2. Pan-Coronavirus Strategies: Broad-Spectrum Immunity Approaches

The pan-coronavirus vaccine represents a significant strategy to address the potential emergence of VOCs. A pan-coronavirus vaccine, also known as a “broadly protective” or “universal” coronavirus vaccine, is a vaccine in development designed to confer extensive immunity against several coronaviruses. The fundamental characteristics of pan-coronavirus vaccines encompass broad-spectrum protection, targeting of conserved areas, and effectiveness against emerging variations [208]. In essence, pan-coronavirus vaccines embody a next-generation strategy designed to surpass the constraints of strain-specific vaccinations by eliciting broader, more enduring immunity against a diverse range of current and prospective coronavirus threats. They are presently in different phases of research and development, with the ultimate objective of their endeavors anticipated to result in proactive pandemic preparedness [209].

A recent review examines the advancement of “universal” coronavirus vaccines designed to offer extensive protection against existing and emerging variants, with the goal of eliminating the necessity for repeated or variant-specific boosters. Numerous vaccine candidates are being developed utilizing mRNA and protein nanoparticle technologies, with various designs targeting distinct regions of the virus, including the spike protein and other viral proteins, to elicit both antibody and cellular immunity. The term “pan-coronavirus” encompasses various definitions, primarily concentrating on sarbecoviruses or betacoronaviruses, rather than achieving comprehensive universal protection. Challenges remain due to immunological uncertainties, despite advancements in the understanding of virus immunology. Moderna’s mRNA-1287 vaccine is designed to target endemic coronaviruses responsible for common colds, rather than to prevent pandemics. Conversely, SK bioscience’s GBP511 candidate employs a “mosaic” strategy incorporating multiple RBDs to achieve extensive B-cell stimulation. The NIH’s pan-coronavirus vaccine program aims to develop both variant-specific and broadly protective platforms, emphasizing ferritin nanoparticle and self-amplifying mRNA technologies [210]. A review article examines heterologous immunity, where immunity to one pathogen affects the response to another, specifically in relation to COVID-19 vaccines and the implications for developing a universal, or “pan-coronavirus”, vaccine. The authors emphasize that the initial COVID-19 vaccines, which primarily target the Spike protein of the original SARS-CoV-2 strain, have provided differing levels of cross-protection against emerging variants. They suggest that comprehending this protection could facilitate the development of universal vaccines. The review highlights the significant role of heterologous (cross-reactive) adaptive immunity in the control of viral spread. This immunity may develop from previous natural infections with related coronaviruses, such as those causing common colds, or from vaccine-induced responses that extend beyond the specific antigen. The review highlights the potential of additional proteins, such as M and N proteins, along with cellular mechanisms, particularly CD4^+^/CD8^+^ T cell responses, in the development of a pan-coronavirus vaccine. T cell responses exhibit greater resilience to viral evolution compared to antibody responses, warranting their consideration. The review indicates that, despite several limitations, first-generation vaccines such as Oxford-AstraZeneca (ChAdOx1nCov-19), Johnson & Johnson (Ad26.COV2.S), Moderna (mRNA-1273), and Pfizer/BioNTech (BNT162b2) induce heterologous immunity to varying extents. The authors conclude that future strategies should emphasize multi-faceted responses, incorporating extensive antibody production, improved cellular immunity, and long-lasting memory, while investigating conserved targets among various coronaviruses [211].

The vaccine’s design is critically important for implementing a pan-coronavirus strategy. A recently published review underscores the significance of design techniques to enhance the efficiency of pan-coronavirus vaccines, along with the hurdles researchers may face in this endeavor. The persistent transmission of SARS-CoV-2, MERS-CoV, the four seasonal HCoVs, and the potential for zoonotic spill-over events underscore the pressing clinical necessity for a pan-coronavirus vaccine. The initial generation of vaccines provided strain-specific antigens. A successful pan-coronavirus vaccine must provide protection against infection, transmission, and illness with enduring, single-dose efficacy, minimal reactogenicity, and universal applicability. The spike protein of SARS-CoV-2 is extremely mutable, necessitating the development of vaccines based on conserved sequences. Developing pan-coronavirus strategies encompasses nanoparticle administration, mosaic antigens, serial vaccination, consensus sequence design, computational antigen design, and multi-antigen vaccines. Future advancements should prioritize robust immunity and mucosal immunity. The difficulties encountered in previous universal vaccination initiatives, particularly for influenza, can provide a framework or guideline in the pursuit of a COVID-specific pan-coronavirus vaccine [208].

Numerous studies have demonstrated encouraging outcomes regarding tactics for the design of pan-COVID vaccines. A recent study assessed an intranasally delivered “pan”-coronavirus vaccine, termed PanCoVac, in Roborovski dwarf hamsters, showing preliminary protection against SARS-CoV-2 infection [212]. PanCoVac comprises conserved T cell epitopes derived from all structural proteins of coronaviruses. A solitary modest dose of NILV-PanCoVac, delivered intranasally to Roborovski dwarf hamsters, decreased viral loads in the lungs and averted infection-related symptoms in the short term (2 dpi), despite the absence of nAbs. Moreover, the protective efficacy of PanCoVac was not contingent upon the generation of nAbs; rather, it appeared to rely on a vigorous T cell response. This work enhances comprehension of universal vaccinations, particularly for mucosal immunity and cellular immune responses.

Cytotoxic T lymphocytes (CTLs), or CD8^+^ T cells, play a vital role in vaccines by destroying virus-infected cells, a fundamental mechanism for regulating viral replication and averting sickness, particularly in instances of chronic infections [213,214]. The capacity of CTLs to regulate viral replication is especially crucial for vaccines targeting viruses that induce persistent infections, such as HIV, hepatitis B, and hepatitis C [215]. Vaccines can provide clinical protection prior to the generation of nAbs, indicating that T cells, such as CTLs, are crucial for early defense [216]. Comprehending the function of CTLs in immunity is crucial for developing successful vaccines, as vaccines can be tailored to provoke robust CTL responses. Vaccines that successfully elicit CTLs, alongside antibodies, yield a more thorough and resilient immune response. CTLs are essential for eradicating cells infected with intracellular pathogens, including viruses, hence potentially providing extensive protection against variations, diminishing illness severity, and fostering enduring immunity. Contemporary vaccine platforms, such as mRNA and viral vectors, are notably effective in eliciting these essential CTL responses [217]. A recent report indicates the potential for employing a rationally designed mosaic antigen in the development of a pan-coronavirus vaccine, emphasizing broad T-cell recognition through conserved CTL epitopes. The research employed a genetic algorithm to create a mosaic antigen encompassing a wide range of conserved CTL epitopes from various coronaviruses, while a panel of human leukocyte antigens (HLAs) was utilized to evaluate potential CTL epitopes. The analysis examined seven coronaviruses that infect humans and their four primary structural proteins: S, M, N, and E. Notably, the mosaic antigen exhibited extensive coverage of CTL epitopes identified in prior studies. The developed mosaic antigen is anticipated to elicit robust cellular immunity by focusing on conserved epitopes present in various coronaviruses. Structural analysis (QMEAN) indicates that the mosaic antigen closely resembles natural viruses and retains the capacity to elicit strong cellular immune responses. The findings indicate the feasibility of targeting conserved CTL epitopes for the development of a pan-coronavirus vaccine [218].

**Figure 1:**
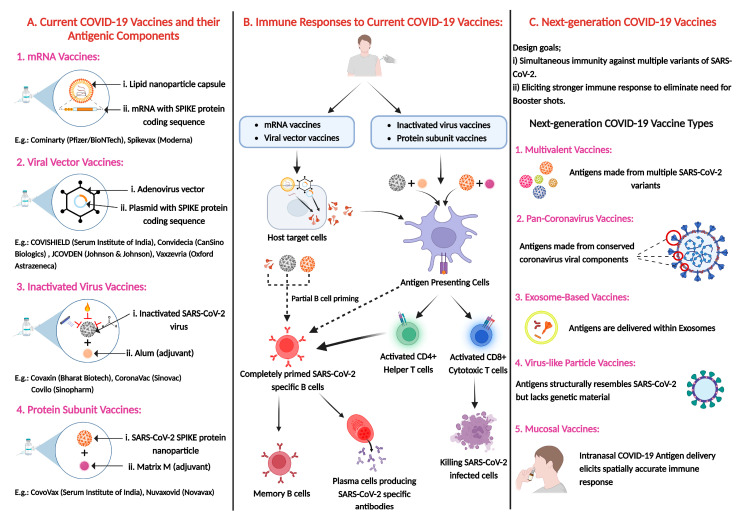
A comprehensive schematic overview of current COVID-19 vaccines, the immune response they elicit, and next-generation vaccine candidates in development. Panel (**A**) outlines the major types of currently used COVID-19 vaccines, including mRNA, viral vector, inactivated, and protein subunit vaccines, along with their core antigenic components. Panel (**B**) illustrates the typical immune response following vaccination, detailing antigen presentation, T cell activation, and B cell-mediated antibody production. Panel (**C**) highlights next-generation COVID-19 vaccines under development, such as multivalent, pan-coronavirus, exosome-based, virus-like particle, and mucosal vaccines, designed to enhance immunity against emerging variants and reduce the need for booster doses (Created with BioRender.com).

A study involving nanoparticles in macaques has identified a promising pan-coronavirus vaccine utilizing a 24-mer SARS-CoV-2 RBD nanoparticle (RBD-scNP), demonstrating broad protective efficacy in animal models. The RBD-scNP vaccine has shown efficacy against multiple coronaviruses, including SARS-CoV-2, SARS-CoV-1, and other bat coronaviruses. The vaccine comprises highly conserved RBD sequences and induces a robust antibody response that neutralizes viral infection. In macaques, it elicited elevated RBD-specific neutralizing antibody titers, surpassing those observed with natural infections or mRNA vaccines. Antibodies derived from vaccinated macaques inhibited the binding of ACE-2 and DH1047 nAbs to SARS-CoV-2, indicating the production of antibodies like DH1047. The vaccine conferred full protection in the respiratory tract of macaques following challenge. The induced antibodies remained unaffected by the mutations found in the emerging SARS-CoV-2 variants B.1.1.7, B.1.351, and P.1. The findings suggest that this candidate is capable of providing protection against future coronavirus outbreaks [219]. The current COVID-19 vaccines, their core antigenic components, and the immune response they elicit have been schematically illustrated in Figure 1. It also highlights next-generation vaccine candidates under development such as multi-valent vaccines and pan-coronavirus strategies designed to enhance immunity against emerging variants and reduce the need for boosters (Figure 1).

## 7. Next-Generation SARS-CoV-2 Vaccines: Addressing the Challenges of Emerging Variants

The forthcoming phase of COVID-19 vaccination initiatives emphasizes the improvement of durability, breadth, and accessibility to effectively respond to the changing landscape of viral variants and the global requirements for immunization. Significant progress has been made with the creation of universal coronavirus vaccines aimed at conserved viral regions, designed to offer protection against various variants and associated coronaviruses. mRNA and protein-based vaccines are undergoing refinement to enhance the duration of immune responses, streamline dosing schedules, and optimize storage conditions, thereby increasing their viability for worldwide distribution. Furthermore, there are ongoing efforts to develop intranasal and oral vaccines aimed at enhancing mucosal immunity and mitigating viral transmission. Initiatives are focused on ensuring fair distribution, utilizing scalable production technologies and cooperative efforts to tackle inequalities in global vaccine accessibility [208,220,221,222,223,224,225,226,227].

### 7.1. Design and Platforms of Next-Generation SARS-CoV-2 Vaccines

Next-generation SARS-CoV-2 vaccines are designed to enhance immune stimulation, improve safety, and simplify distribution [228]. This section provides an overview of key next-generation vaccine platforms under development (Table 3).

#### 7.1.1. Exosome-Based Vaccines

Exosomes are small extracellular vesicles released by various cell types, playing a crucial role in intercellular communication by transporting proteins, lipids, and RNA. Their natural ability to encapsulate and deliver biomolecules makes them a promising platform for cell-free vaccine delivery. Unlike traditional vaccines, exosome-based vaccines present antigens in a way that elicits strong immune responses without requiring live pathogens [250,251]. Exosome-based vaccines efficiently incorporate and protect antigenic molecules, facilitating their uptake by antigen-presenting cells (APCs). Studies have shown that exosomes effectively present antigens to dendritic cells, leading to robust activation of both cellular and humoral immunity [252]. Notably, exosomes promote antigen cross-presentation, stimulating cytotoxic T-cell responses critical for antiviral defense. Additionally, they can be engineered to carry immunostimulatory molecules, enhancing their adjuvant properties and further amplifying immune activation [253]. Preclinical studies are evaluating exosome-based vaccines engineered to deliver viral antigens from influenza, HIV, and coronaviruses [254]. Kim et al. demonstrated an exosome-based SARS-CoV-2 vaccine approach designed to deliver structural protein antigens, effectively eliciting robust CD8^+^ T cell and B cell responses [255]. Similarly, Cacciottolo et al. demonstrated the use of an exosome-based platform for a bivalent SARS-CoV-2 vaccine expressing the Delta variant spike (STX-S) and nucleocapsid (STX-N) proteins. When administered individually or in combination (STX-S + N), this vaccine induced strong humoral and cellular immune responses in mice and rabbits, even at nanogram doses without the need for an adjuvant. The StealthX exosome platform further exhibited the ability to generate potent nAbs with cross-reactivity to diverse spike variants while also eliciting strong T cell responses [237]. In addition to injectable formulations, an inhalable vaccine utilizing lung-derived exosomes conjugated with recombinant SARS-CoV-2 RBD demonstrated thermal stability and effectively induced both systemic and mucosal immunity in mice. This formulation elicited RBD-specific IgG, mucosal IgA, and Th1-biased CD4^+^ and CD8^+^ T cell responses, providing a promising needle-free and logistically viable alternative for global immunization efforts [238].

#### 7.1.2. Virus-like Particle (VLP) Vaccines

Virus-like particles (VLPs) resemble viruses structurally but lack genetic material, making them inherently non-infectious and safe. By mimicking the outer architecture of pathogens, VLPs effectively stimulate strong and durable immune responses. This technology has been successfully employed in existing vaccines for hepatitis B and human papillomavirus (HPV) [256,257]. During the COVID-19 pandemic, VLP-based vaccines were developed against SARS-CoV-2, incorporating key viral proteins, such as the spike protein, to replicate the virus’s native structure. Preclinical studies have demonstrated that these vaccines elicit high levels of nAbs, suppress viral replication, and exhibit fewer adverse effects compared to other vaccine platforms. Additionally, advances in scalable production methods using plant and insect cell systems have enhanced the feasibility of large-scale VLP vaccine manufacturing [258,259]. Beyond SARS-CoV-2, VLP-based vaccine research is expanding to other infectious diseases, including norovirus, Zika virus, and dengue, where traditional vaccine approaches have shown variable efficacy [260].

#### 7.1.3. Mucosal Vaccines

Mucosal immunity provides broad protection at key pathogen entry sites, including the respiratory and gastrointestinal tracts. However, conventional injectable vaccines often fail to elicit strong immune responses at these mucosal barriers, limiting their ability to prevent infection and transmission. Mucosal vaccines aim to overcome this limitation by inducing both local and systemic immune responses at the site of infection [261]. Mucosal vaccines elicit protective immunity by delivering antigens to mucosa-associated lymphoid tissues (MALTs), where specialized APCs, including microfold (M) cells and dendritic cells, facilitate antigen uptake and processing. This localized antigen presentation induces robust mucosal immune responses, characterized by the production of secretory IgA (sIgA) and the activation of tissue-resident T cells, thereby enhancing both humoral and cellular immunity [262]. Next-generation mucosal vaccines, administered via nasal or oral routes, further optimize mucosal immunity by stimulating the production of sIgA antibodies, which neutralize pathogens at mucosal surfaces, limiting their transmission and providing protection against both symptomatic and asymptomatic infections [263]. Intranasal SARS-CoV-2 vaccines have shown promise in preclinical studies by lowering viral loads in the upper respiratory tract and blocking viral transmission [264]. Oral mucosal vaccines are also being explored for diseases such as cholera and rotavirus, with encapsulation technologies improving antigen stability against digestive degradation, enhancing immunogenicity. Additionally, mucosal vaccines offer needle-free administration, which could improve vaccine accessibility and acceptance, particularly in resource-limited settings [265,266].

#### 7.1.4. Nanomaterial-Based Vaccines

Nanotechnology has emerged as a critical component in the development of SARS-CoV-2 vaccines, particularly through its role in designing and delivering next-generation vaccine platforms. The remarkable success of mRNA vaccines, such as BNT162b2 and mRNA-1273, is largely attributed to LNPs, which protect mRNA from enzymatic degradation while facilitating efficient cellular uptake and cytoplasmic release, thereby ensuring robust antigen expression [117,267]. Beyond LNPs, alternative nanoparticle-based strategies, including polymeric nanoparticles and virus-like particles, are being explored to improve antigen stability and enable targeted delivery to APCs. These approaches support controlled antigen release, ultimately enhancing the magnitude and durability of immune responses [268]. Additionally, nanoparticle technology is being leveraged to present the SARS-CoV-2 spike protein and its conserved domains in multivalent structures, mimicking viral structures to optimize B cell activation and affinity maturation [269,270]. Furthermore, novel adjuvant systems incorporating nanomaterials are now being integrated into protein- and peptide-based vaccines to enhance both mucosal and systemic immune responses. These nanoscale adjuvants function as antigen depots at the injection site, prolonging antigen availability and promoting the establishment of durable immune memory [271].

### 7.2. Innovations in Vaccine Delivery Systems

Recent advancements in vaccine technology have led to the development of sophisticated delivery platforms that enhance vaccine efficacy, stability, and accessibility. Among these, nanoparticle-based and microneedle systems have emerged as transformative alternatives to conventional injection methods [272]. Nanoparticles play a crucial role in modern vaccine delivery, particularly in the success of mRNA vaccines against COVID-19. LNPs protect mRNA from enzymatic degradation, facilitate efficient cellular uptake, and enhance antigen presentation by APCs [273]. Additionally, nanoparticle-based systems are being explored for the targeted delivery of protein and DNA vaccines, offering controlled antigen release and improved immune activation [272,274,275]. Microneedles represent another groundbreaking advancement, enabling painless, minimally invasive vaccine administration through the skin. These microneedle arrays deliver antigens directly to immune-rich skin layers, inducing rapid and localized immune responses. Their ease of storage, minimal refrigeration requirements, and potential for self-administration make them particularly suited for large-scale vaccination efforts in remote and resource-limited regions [276]. Further innovations include electroporation, which enhances DNA vaccine uptake by temporarily increasing cell membrane permeability, and polymer-based delivery systems designed to improve antigen stability, reduce dose requirements, and strengthen both systemic and mucosal immune responses [277]. Ongoing research in these advanced delivery systems is poised to revolutionize vaccine administration, making it more effective, accessible, and user-friendly.

### 7.3. Next-Generation Vaccines: Advancing Broader Immunological Protection and Durability

Next-generation vaccines are designed to elicit stronger and longer-lasting immune responses while mitigating severe disease. These vaccines address critical challenges such as pathogen mutations, immune evasion, and waning immunity—factors that have compromised the effectiveness of conventional vaccines. Achieving broad and durable immune protection is essential for combating rapidly evolving viruses like influenza, and SARS-CoV-2 [278]. Key advancement in next-generation vaccines is their ability to confer immunity against multiple strains, variants, and related pathogens. Traditional vaccines often target antigenic regions prone to mutation, leading to reduced efficacy against emerging variants. In contrast, novel vaccine platforms prioritize conserved epitopes that remain stable despite viral evolution. For instance, mRNA and protein subunit vaccines incorporate epitopes from various viral proteins, including conserved regions of the SARS-CoV-2 spike protein, eliciting cross-reactive T-cell and antibody responses. A recent review highlights that incorporating viral components beyond the essential S protein, such as the Nucleocapsid protein, may provide significant opportunities for the advancement of next-generation vaccines. The nucleocapsid protein, an essential element of the SARS-CoV-2 virus, has been recognized as a promising candidate for next-generation vaccines. This protein is essential for replication and immune responses and may improve efficacy and protection against multiple viruses, including their variants. The integration of nucleocapsid and spike proteins may elicit robust and long-lasting immunity, thereby enhancing the efficacy of vaccination efforts and bolstering the ability to effectively address COVID-19 [222]. This strategy enhances protection against multiple variants by effectively targeting mutated antigenic sites [76,279,280]. Similarly, VLP vaccines mimic the structural complexity of native viruses, improving recognition by both B and T cells [260,281]. Adjuvants also play a pivotal role in amplifying immune responses. Novel adjuvants, such as Toll-like receptor agonists and saponins, enhance innate immune activation, promote antigen-specific T cell responses and robust antibody production by B cells. These adjuvants ensure a balanced immune response, preventing immune skewing while fostering strong, and multi-target protection [282,283].

Durability is a crucial determinant of vaccine-induced immunity, influencing the need for booster doses. Long-lasting protection is particularly vital for diseases requiring sustained immunity, such as measles, hepatitis, and COVID-19. While vaccines against influenza and coronaviruses exhibit waning effectiveness, necessitating periodic boosters, innovative strategies are being explored to extend immune memory [284,285]. One promising approach involves nanoparticle-based vaccines, which create antigen depots at injection sites, leading to prolonged antigen release and facilitating the generation of long-lived plasma cells and memory T cells [286,287]. Additionally, targeting follicular helper T (Tfh) cell activation has been shown to enhance germinal center responses, promoting B cell affinity maturation and the production of high-affinity, long-lasting antibodies. Studies on mRNA vaccines have demonstrated strong Tfh cell activation, supporting durable plasma cell development and sustained antibody production [288]. Both humoral and cellular immunity are essential for establishing comprehensive and long-lasting protection. Neutralizing antibodies block viral entry and prevent early infections; however, antibody levels naturally decline over time, reducing their protective efficacy [15]. In contrast, memory CD4^+^ and CD8^+^ T cells provide a secondary defense by recognizing and eliminating infected cells, contributing to durable immunity [289,290]. Studies suggest that vaccines capable of eliciting strong T-cell immunity remain effective even as viral antigens evolve [291,292].

Next-generation vaccines aim to maximize efficacy by enhancing both humoral and cellular responses. DNA and mRNA vaccines, for example, induce potent cytotoxic CD8^+^ T-cell responses by directing antigen expression within host cells, ensuring efficient presentation on MHC class I molecules—an essential mechanism for controlling intracellular viral replication [241,293]. By integrating innovative strategies—including conserved antigen targeting, adjuvant enhancement, nanoparticle-based antigen delivery, and optimized booster schedules—next-generation vaccines offer promising solutions to the challenges posed by rapidly mutating viruses. These advancements pave the way for stronger and more durable immune protection, ensuring the continued efficacy of vaccines in the face of emerging viruses.

### 7.4. Next-Generation Vaccine Efficacy Against Emerging Sub-Variants

Next-generation vaccines have demonstrated efficacy against newly emerged SARS-CoV-2 sub-variants. The continuous evolution of the virus has led to the emergence of multiple sub-variants with enhanced transmissibility and immune evasion capabilities. Notable variants include BA.2-derived BJ.1 and BM.1.1.1, XBB lineages such as XBB.1.9, XBB.1.5, and XBB.1.16, as well as the highly mutated BA.2.86 (Pirola) variant. To counter these adaptations, researchers are refining next-generation vaccines to target critical viral antigenic sites. This section summarizes the efficacy data of these vaccines against recently identified sub-variants.

#### 7.4.1. BA.2-Derived Sub-Variants: BJ.1 and BM.1.1.1

BJ.1 and BM.1.1.1, originating from the BA.2 lineage, harbor mutations in the spike protein’s RBD that may enhance immune evasion. Early studies suggest that these mutations reduce the neutralization efficacy of vaccines designed against earlier SARS-CoV-2 variants. However, mRNA-based vaccines targeting Omicron sub-lineages continue to exhibit significant cross-neutralizing activity [19]. Ongoing clinical trials and real-world studies are assessing the effectiveness of updated vaccines against these emerging sub-variants. Preliminary data indicate that booster doses incorporating BA.5 and XBB.1.5 antigens provide protection against severe disease and hospitalization caused by BJ.1 and BM.1.1.1 [206,294].

#### 7.4.2. XBB Lineages: XBB.1.9, XBB.1.5, and XBB.1.16

The XBB lineage emerged through the recombination of two Omicron sub-variants (BA.2.10 and BA.2.75) and carries mutations that enhance immune evasion. Among these, XBB.1.5, known as the “Kraken” variant, rapidly became a dominant strain worldwide due to its high transmissibility and partial resistance to nAbs [295]. Similarly, XBB.1.9 and XBB.1.16 have established dominance in various regions globally [296]. Next-generation vaccines targeting XBB.1.5 have demonstrated strong efficacy. mRNA vaccines incorporating the XBB.1.5 spike protein elicit significant increases in neutralizing antibody levels against multiple XBB sub-lineages, including XBB.1.9 and XBB.1.16 [297]. Booster doses with this formulation induce neutralizing antibody responses comparable to those observed against earlier Omicron strains, highlighting their cross-protective potential [298]. Furthermore, real-world data confirm that updated vaccines provide strong protection against severe disease and hospitalization in regions where XBB variants are prevalent. Ongoing research also suggests that T-cell responses remain robust despite spike protein mutations, contributing to sustained immunity [195,299].

#### 7.4.3. BA.2.86 (Pirola) Sub-Variants

The BA.2.86 (Pirola) variant, first identified in mid-2023, attracted scientific attention due to its exceptionally high number of mutations in both spike and non-spike regions [300]. These mutations raise concerns about enhanced immune evasion and potential reductions in vaccine efficacy. However, early research indicates that next-generation Omicron-targeted vaccines provide moderate to strong protection against BA.2.86 [301]. Moderna and Pfizer have reported that their latest mRNA vaccines elicit strong neutralizing responses against BA.2.86, with booster doses significantly increasing neutralizing antibody levels compared to pre-booster levels [302]. Similarly, Novavax’s updated protein-based vaccine has demonstrated robust immune responses against Pirola and its related sub-variants [303,304]. Ongoing studies are assessing the durability of this protection. Preliminary findings suggest that memory B cells generated by prior Omicron-targeted vaccines can adapt to recognize BA.2.86 and produce high-affinity antibodies upon re-exposure [305].

### 7.5. Enhancing Cross-Variant Immunity and Immune Memory Through Next-Generation and Heterologous Vaccination Strategies

Achieving broad and durable immunity against SARS-CoV-2 requires innovative vaccination approaches that address the challenges posed by emerging variants. Next-generation vaccines, designed to target conserved viral antigens, have shown potential in eliciting cross-variant protection by inducing nAbs and robust memory B and T cell responses [306]. These vaccines leverage advancements in antigen design, adjuvants, and delivery systems to extend the duration of immunity. Research highlights the importance of immune memory, with studies demonstrating that while neutralizing antibody levels fluctuate across variants, long-term protection relies on sustained memory responses [307,308,309]. Novel vaccine formulations, including those tailored for Omicron sub-variants (e.g., BJ.1, BM.1.1.1, and XBB lineages), have maintained protective efficacy by inducing cross-reactive cellular immunity despite antigenic drift [310].

Heterologous vaccination, or “mix-and-match” strategies, further enhance cross-variant immunity by combining different vaccine platforms, such as mRNA, viral vector, and protein-based formulations. This approach has been shown to generate broader and more durable immune responses compared to homologous regimens, increasing both neutralizing antibody titers and T cell activation [311,312]. Different vaccine platforms elicit immune responses through distinct mechanisms. For example, mRNA vaccines (e.g., Pfizer-BioNTech, Moderna) encode spike protein antigens, inducing both humoral and cellular immunity [115]. Whereas viral vector vaccines (e.g., AstraZeneca, Johnson & Johnson) use modified viruses to deliver spike protein genes, activating immune pathways through endogenous antigen presentation [313]. On the other hand, Protein-based vaccines (e.g., Novavax) directly present spike proteins or virus-like particles to APCs, promoting strong B-cell activation [314]. Heterologous vaccination, such as a viral vector vaccine (such as AstraZeneca) followed by an mRNA booster (Pfizer or Moderna), enhances antigen recognition, improves memory B and T cell activation, and increases neutralizing antibody titers compared to homologous regimens, likely due to differential antigen presentation mechanisms [315,316]. A follow-up study found that individuals who received a viral vector prime followed by an mRNA booster maintained strong antibody and T-cell responses for over a year with minimal decline in protection against severe disease, suggesting that heterologous vaccination may reduce the need for frequent boosters [317]. Studies indicate that individuals receiving heterologous booster doses exhibit superior protection against immune-evasive variants like Delta and Omicron [318,319]. Similarly, emerging evidence suggests that combining mRNA-based vaccines with protein-based or nanoparticle-based boosters enhances cross-neutralization against newly circulating variants such as XBB and BA.2.86 [302,310].

Beyond immunogenic benefits, heterologous strategies offer practical advantages, including improved vaccine accessibility and reduced dependency on specific platforms. This approach has been instrumental in addressing supply constraints and mitigating rare adverse effects associated with certain vaccine types [320,321]. Additionally, personalized booster regimens based on immune profiling may further refine vaccination strategies, optimizing individual protection by reducing the risk of complications associated with specific vaccine platforms [322,323]. As SARS-CoV-2 continues to evolve, integrating next-generation vaccine technologies with heterologous booster strategies holds promise for sustaining long-term immunity and enhancing preparedness against future variants. Heterologous vaccination strategies, despite their advantages, face challenges related to regulatory approval, as comprehensive data are required to validate mixed-dose regimens, delaying implementation. Ongoing research is needed to determine optimal vaccine combinations and dosing intervals, as immune responses vary based on vaccine type and order of administration [324,325]. Logistical hurdles include ensuring healthcare providers and the public are well-informed about the safety and efficacy of these regimens. Clear communication is crucial to building trust and addressing concerns about vaccine effectiveness, particularly in communities unfamiliar with mixed-dose strategies [326].

### 7.6. Immune Imprinting and Next-Generation Vaccines

The immune system exhibits immune imprinting (also known as original antigenic sin), wherein memory responses generated from prior pathogen encounters can limit its ability to recognize and respond effectively to novel viral variants. This phenomenon poses a significant challenge for vaccine development against rapidly mutating viruses such as influenza and SARS-CoV-2 [327]. Next-generation vaccines aim to overcome immune imprinting through two primary strategies: targeting conserved viral protein regions and broadening the spectrum of antigenic targets in vaccine formulations. Advanced mRNA vaccine designs incorporate instructions for expressing multiple viral epitopes, including both conserved and variable regions. Recent studies indicate that these vaccines elicit a well-balanced immune response, generating both cross-reactive and variant-specific antibodies. By mitigating immune imprinting, these vaccines enhance protection against emerging variants while potentially reducing the frequency of booster doses required for sustained immunity [328,329]. Ongoing research also focuses on the development of novel adjuvant systems designed to modulate immune responses, thereby extending immune coverage and durability. These approaches leverage Toll-like receptor (TLR) agonists and other immune activators to shift immune responses away from reliance on pre-existing memory cells [330]. Next-generation vaccine platforms offer promising solutions to challenges associated with traditional vaccines. Advances in biotechnology, immunology, and nanotechnology are driving the development of more effective and accessible vaccines, ensuring improved protection against both current and emerging infectious threats [331].

## 8. Discussion: Challenges and Future Perspectives

The swift development and deployment of SARS-CoV-2 vaccines represent a landmark achievement in modern medical research. Within the first year of the COVID-19 pandemic, multiple vaccines received emergency use authorization and demonstrated high efficacy in reducing severe disease, hospitalizations, and mortality. The global vaccination effort was driven primarily by mRNA-based vaccines (Pfizer-BioNTech, Moderna) and viral vector vaccines (AstraZeneca, Johnson & Johnson), while protein subunit vaccines (Novavax) and inactivated virus vaccines (Sinovac, Sinopharm) also played crucial roles [117,332]. The pandemic underscored the need for rapid technological advancements, global collaboration, and equitable vaccine distribution to counter ongoing viral mutations and emerging variants. However, challenges such as vaccine hesitancy, inequitable access, immune escape, and waning immunity hindered sustained disease control [333,334]. Vaccine hesitancy remains a major barrier to global immunization efforts, driven by misinformation, distrust in healthcare systems, and concerns about vaccine side effects. Addressing vaccine hesitancy requires sustained, transparent communication strategies, collaboration with trusted community leaders, and culturally tailored public health campaigns to mitigate concerns and improve acceptance [335,336,337].

Beyond hesitancy, vaccine equity remains a persistent global challenge. During the early phase of the pandemic, high-income countries secured large vaccine supplies, while low- and middle-income countries faced severe delays in procurement and distribution. The need for ultra-cold storage for mRNA vaccines further exacerbated disparities, as many regions lacked the necessary infrastructure to maintain proper storage conditions, particularly in remote areas. COVAX was established to facilitate equitable vaccine distribution, aiming to provide access to underserved populations through procurement and logistical support [338]. However, structural inequities continue to impede progress. In response, researchers and manufacturers are developing next-generation vaccines with improved thermostability, reduced storage requirements, and alternative delivery methods such as intranasal and oral formulations, which may enhance accessibility and global immunization efforts [339]. Effective vaccination programs must also be integrated with broader public health strategies to maximize impact [340]. Outbreak prevention requires more than immunization alone; genomic surveillance, widespread testing, and early intervention strategies are critical for monitoring viral evolution and adapting vaccination protocols accordingly. A comprehensive approach that combines vaccination with robust infectious disease control measures can optimize healthcare resource allocation, particularly in regions with limited medical infrastructure [341].

The emergence of SARS-CoV-2 VOCs, including Delta, Omicron, and their sub-lineages, such as XBB, JN.1, and BA.2.86, has underscored the limitations of first-generation COVID-19 vaccines in providing broad and durable protection against evolving viral variants. The mutations in these variants enable them to partially evade immune defenses, resulting in reduced vaccine efficacy against infection and mild disease. The continuous emergence of new variants underscores the need for ongoing surveillance and prompt adaptation of vaccine formulations. Research indicates that while nAb levels decline months after vaccination, long-term protection against severe disease relies more on the sustained activity of memory B and T cells, as well as other cellular immune responses [342,343,344]. Consequently, vaccine development should prioritize strategies that ensure durable humoral and cellular immunity over extended periods. An effective strategy to enhance and sustain immune memory involves optimizing the timing and formulation of booster vaccinations. While booster doses are designed to prolong protection by reactivating immune memory, the durability of this immunity can wane over time, necessitating additional doses to maintain adequate defense. Moreover, the emergence of novel viral variants may reduce the effectiveness of existing booster formulations, particularly when antigenic drift leads to immune escape. Recent studies suggest that personalized booster schedules, incorporating controlled re-exposure to variant-specific antigens, can significantly improve the quality of immune recall responses. Such approaches have been shown to elicit more rapid and robust immune responses upon reinfection [345,346]. However, a key yet often overlooked barrier to booster dose uptake is vaccine hesitancy stemming from adverse reactions experienced during the initial vaccine doses. Although only a minority of vaccine recipients report moderate to severe side effects, these experiences contribute to vaccine hesitancy and reduce the willingness to receive additional doses [347,348]. This decline in booster dose acceptance poses a substantial challenge to public health efforts, particularly as maintaining protection against emerging immune-evasive variants relies heavily on booster-induced immunity [349]. Addressing this barrier requires clear, evidence-based communication strategies that help the public differentiate between common, mild side effects and rare but serious adverse events. Furthermore, transparent safety monitoring systems, supported by rigorous data analysis, are essential to build public trust [350]. In parallel, the development of next-generation vaccines with improved safety profiles and greater tolerability offers a promising avenue to increase booster acceptance among hesitant populations, thereby supporting broader immunization coverage and sustained pandemic control.

Neutralizing antibodies play a crucial role in controlling SARS-CoV-2 infections, initially showing effectiveness against early variants. However, highly mutated variants, such as Omicron, have demonstrated substantial antibody escape, reducing the ability of vaccine-induced neutralization. New vaccines and adaptive strategies have partially addressed these challenges, with booster doses increasing effectiveness [351]. Monoclonal antibody (mAb) therapies were critical in early COVID-19 management, especially for vulnerable populations. Early agents like Bamlanivimab and Casirivimab/Imdevimab significantly reduced severe symptoms. However, mutations in the spike protein, seen in variants such as Delta and Omicron, diminished the effectiveness of these first-generation mAbs, leading to their removal from the market [352,353]. In response to evolving variants, researchers are developing next-generation mAbs that target conserved regions of the virus, such as the spike protein’s fusion peptide. These novel therapies may be more effective against variants like XBB sub-lineages and potentially offer longer-lasting protection. Researchers are also exploring combinations of multiple epitopes to reduce the risk of escape mutations. In the long term, broad-spectrum mAbs targeting conserved viral regions could provide durable protection against SARS-CoV-2 and other coronaviruses. These advancements, alongside responsive vaccines, are expected to improve pandemic resilience and counter future viral threats.

The development of next-generation vaccines remains a key priority to address the ongoing challenges posed by SARS-CoV-2. Multivalent vaccines, which incorporate antigens from multiple variants, are being explored to offer immunity against both current and emerging strains, potentially reducing the need for frequent booster shots. Additionally, studies are investigating the inclusion of viral elements, such as the nucleocapsid protein, to enhance long-term immune responses [354]. Efforts are also underway to create universal vaccines targeting stable viral regions, which could provide extended protection against various coronaviruses [206]. Wang et al. engineered multiple subunit vaccines using a conserved spike backbone from different SARS-CoV-2 Delta and Omicron subvariants and assessed the immunogenicity and cross-protective potential of these constructs. Among these, RBDs from the Delta variant (S-6P-Delta-RBD) induced robust and broad nAbs against multiple SARS-CoV-2 variants, including Omicron subvariants BA.1, BA.2, BA.2.75, BA.4.6, and BA.5 [355]. Another promising area of research is the optimization of mucosal vaccines, which can deliver local immunity at critical entry sites such as the respiratory tract. These vaccines could reduce infection rates, limit transmission, and strengthen community-wide protection [266]. Together, these strategies aim to minimize the need for continuous vaccine updates while fostering robust immunity across populations.

SARS-CoV-2 infection induces significant epigenetic modifications in host cells, facilitating viral persistence, immune evasion, and disease progression [356]. Critical antiviral genes are suppressed through epigenetic modifications, including histone acetylation changes and DNA methylation alterations, impairing interferon signaling and immune response pathways [357,358]. Understanding the interplay between epigenetic reprogramming and sustained immune memory could drive the development of more effective vaccines with prolonged protection. Additionally, noncanonical nucleic acid structures, G-quadruplexes (G4s), have emerged as potential antiviral targets in SARS-CoV-2, with several G4 ligands identified for their role in modulating viral replication and pathogenesis [359,360,361,362,363]. Further investigation into G4-mediated immune evasion and viral adaptation may provide crucial insights for next-generation antiviral strategies.

The knowledge gained from the COVID-19 pandemic provides valuable insights for enhancing future pandemic preparedness. The rapid development and global distribution of vaccines during the pandemic underscored the critical importance of worldwide cooperation and advanced technological platforms. Decades of research on influenza antigenic drift have highlighted the need for ongoing surveillance and periodic vaccine reformulations to address viral evolution. These strategies were reinforced with the emergence of SARS-CoV-2 VOCs, with genomic surveillance playing a pivotal role in detecting and characterizing new variants. To better prepare for future health emergencies, public health systems must strengthen genomic surveillance capabilities, enhance manufacturing infrastructure, and establish rapid vaccine distribution protocols [364]. Ongoing surveillance, combined with timely adjustments to vaccination strategies, is crucial to address the evolving nature of SARS-CoV-2. Genomic sequencing and real-time data collection, along with variant tracking, provide critical insights into how new mutations affect vaccine efficacy [365]. Public health authorities must also ensure the rapid development of adaptable vaccination protocols that can respond to new scientific evidence.

Research into vaccine adjuvants, delivery systems, and alternative dosing schedules remains essential to ensure sustained immune protection across diverse population groups. Moving forward, infectious disease management must be both proactive and collaborative. To mitigate the impacts of future pandemics, global health systems should be strengthened through comprehensive vaccination programs, effective disease surveillance, and robust healthcare infrastructure. Global cooperation must focus on the equitable distribution of healthcare resources, particularly in regions most vulnerable to infectious outbreaks. Despite progress in vaccine development and deployment, ongoing challenges—such as vaccine hesitancy, global inequities, and the emergence of new viral variants—demonstrate why sustained research and innovation remain critical. By learning from the COVID-19 pandemic’s challenges, the global community can build stronger health systems capable of protecting populations from future infectious diseases.

## 9. Conclusions

The COVID-19 pandemic catalyzed the fastest vaccine development effort in modern history, leading to the deployment of first-generation vaccines that significantly reduced disease severity, hospitalizations, and mortality [366]. These vaccines, primarily targeting the SARS-CoV-2 spike protein, successfully induced systemic IgG responses but demonstrated limited efficacy in preventing infection and transmission, especially in the context of rapidly emerging variants [367,368,369]. This limitation is partly due to waning humoral immunity and insufficient induction of mucosal immune memory, as intramuscular delivery predominantly elicits systemic IgG responses without generating robust mucosal IgA antibodies required for viral neutralization at the respiratory entry sites [370,371]. Moreover, SARS-CoV-2’s high mutation rate, particularly in the spike protein, allows it to evade neutralizing antibodies, contributing to breakthrough infections and reinfections [372,373]. While antibody responses decline over time, T cell-mediated immunity—especially CD8^+^ cytotoxic T cells—offers greater resilience and cross-reactivity against emerging variants [374,375,376]. Nevertheless, current vaccines primarily target the spike protein and do not fully engage the breadth of cellular immunity, which limits their efficacy against antigenically distinct variants like Omicron [305,377]. Repeated booster doses that focus solely on the spike antigen may yield diminishing returns unless they expand the immune repertoire to include more conserved viral components [378,379]. Therefore, next-generation vaccine strategies should aim to stimulate both systemic and mucosal immunity by incorporating conserved viral antigens—such as nucleocapsid (N), membrane (M), or envelope (E) proteins—alongside the spike protein, to enhance cross-variant protection [380,381]. Intranasal vaccines employing live attenuated viruses or viral vectors offer promising prospects by simultaneously eliciting mucosal IgA, systemic IgG, and T cell responses, thereby preventing both infection and transmission [32,263,382,383]. Furthermore, innovative adjuvants that activate dendritic cells, promote cytokine secretion, and facilitate cross-presentation to CD8^+^ T cells can enhance cellular responses and establish durable immunity [283]. Additionally, the implementation of novel delivery systems such as nanoparticles or exosome-like vehicles improve antigen presentation, lymph node targeting, and promote more efficient adaptive immune priming [274,275,384]. While traditional platforms such as live attenuated and inactivated vaccines offer strong, long-lasting humoral and cellular immune responses, they pose safety and logistical challenges [385]. In contrast, mRNA and viral vector vaccines offer rapid manufacturing and robust initial immunity but exhibit declining efficacy against antigenically drifted variants over time [386,387]. Their inability to elicit sufficient mucosal immunity—via secretory IgA and tissue-resident memory T cells—limits their role in curbing transmission [388,389]. Future vaccine design must integrate immunological insights with technological innovations—such as self-amplifying RNA, conserved antigen selection, next-generation adjuvants, and advanced delivery platforms—to overcome current limitations. A comprehensive understanding of immune correlates of protection, supported by rigorous preclinical and clinical evaluations, will be critical in guiding the development of next-generation vaccines that provide durable, broad-spectrum protection against evolving SARS-CoV-2 variants and potential future pandemics.

## Figures and Tables

**Table 1:** Key characteristics of SARS-CoV-2 variants of concern: mutations, virulence, immune evasion, and global impacts.

Variants of Concern (VOCs)	Country/First Appearance	Key Mutations	Virulence	Immune Evasion	Global Impact	Most Effective Vaccines	References
Alpha (B.1.1.7)	UK, September 2020	N501Y, P681H, Deletion 69–70	Increased transmissibility, similar severity	Minimal, reduced neutralization by some antibodies	Dominated Europe and North America; increased case numbers	Pfizer-BioNTech, Moderna (effective with high neutralization)	[47,48]
Beta (B.1.351)	South Africa, May 2020	E484K, K417N, N501Y	Moderate, immune escape raises concerns	Moderate, reduced vaccine efficacy	Severe second wave in South Africa; vaccine efficacy challenges	Johnson & Johnson, mRNA vaccines (reduced efficacy for mild cases, good for severe disease)	[49,50]
Gamma (P.1)	Brazil, November 2020	E484K, K417T, N501Y	Moderate, severe outbreaks in seroprevalent regions	Moderate, reinfections in high seroprevalence areas	Regional crises in Brazil and Japan; limited global spread	CoronaVac (moderate efficacy), mRNA vaccines (boosters improve protection)	[51,52]
Delta (B.1.617.2)	India, October 2020	L452R, P681R, D614G	High, increased severity and hospitalization	Minimal to moderate, partially vaccine-resistant	Global dominance; severe outbreaks in India and US	mRNA vaccines with booster doses, AstraZeneca (good for severe disease)	[47,53,54]
Omicron (B.1.1.529)	South Africa, November 2021	E484A, G446S, Deletion 69–70	Reduced intrinsic virulence, high transmissibility	High, reinfections and breakthrough cases common	Massive surges globally; ongoing evolution with sub-lineages	Bivalent mRNA vaccines (targeting Omicron sub-lineages), Booster doses critical	[46,55]

**Table 2:** Efficacy of vaccines, neutralizing antibodies, and monoclonal antibodies against SARS-CoV-2 variants of concern (VOCs).

Variants of Concern (VOCs)	Vaccines	Neutralizing Antibodies (nAbs)	Monoclonal Antibodies (mAbs)	References
Alpha(B.1.1.7)	High efficacy with mRNA vaccines (Pfizer-BioNTech, Andover, MA, USA;Moderna, Norwood, MA, USA); AstraZeneca effective	Minimal reduction; effective neutralization by vaccine and natural infection antibodies	Retained efficacy; mutations did not affect primary binding sites	[47,48]
Beta(B.1.351)	Moderate decrease in efficacy; mRNA vaccines better than vector-based vaccines	Significant reduction; immune escape via E484K mutation	Reduced efficacy for some monoclonal therapies; mutations altered key epitopes	[49,50]
Gamma(P.1)	Reduction similar to Beta; mRNA vaccines effective for severe disease	Moderate to significant reduction; reinfections observed	Reduced neutralizing activity; some therapies less effective	[51,52]
Delta(B.1.617.2)	Efficacy against infection reduced, strong protection against severe disease with boosters	Modest reduction, protection against severe outcomes maintained	Most treatments effective, though some showed reduced potency	[47,157]
Omicron(B.1.1.529)	Significant reduction in protection, strong efficacy against severe disease after boosters or updated vaccines	Substantial decrease in neutralization; reinfections and breakthrough cases common	Significant reduction or loss of efficacy for many therapies; updates needed	[46,55]

**Table 3:** Overview of Next-Generation Vaccine Platforms: Summary of various next-generation vaccine platforms, highlighting their antigen targets, mechanisms of action, formulation strategies, efficacy based on clinical or preclinical outcomes, safety profiles, secondary effects, and known limitations.

Vaccine Type	Examples	Antigen Target	Mechanism of Action	Formulation Type	Efficacy (Preclinical/Clinical)	Notable Secondary Effects	Limitations	References
mRNA-Based	mRNA-1283	Full-length Spike or RBD	Encodes viral protein; translated in host cells to induce humoral and cellular immunity	Lipid nanoparticle	High antibody titers; improved thermal stability (preclinical)	Injection site pain, fatigue, mild fever	Data largely preclinical; cold-chain still needed	[229,230]
Live Attenuated	FluMist, COVI-VAC, BK2102 (experimental)	Whole virus	Uses replication-competent but weakened virus to induce robust immunity	Intranasal spray or oral drops	Strong mucosal and systemic immunity; effective in animal models (preclinical studies)	Nasal irritation, mild symptoms; caution in immunocompromised	Reversion risk; cold chain and safety in immunocompromised	[231,232]
Self-Amplifying RNA (saRNA)	ARCT-154, VLPCOV-01	Spike variants	Intracellular RNA amplification enhances antigen expression and immune response	Lipid nanoparticle	Promising immunogenicity; dose-sparing (Phase I/II trials)	Tolerable; less reactogenic than standard mRNA	Durability and dose optimization under evaluation	[233,234]
Intranasal/Inhaled	BBV154, AdCOVID, VXA-CoV2-1, iNCOVACC	Spike	Induces mucosal IgA and tissue-resident T cell responses	Nasal spray, aerosol, or drops	Shown to reduce nasal viral load; blocks transmission (animals)	Nasal congestion, mild irritation (early trials)	Variable immunogenicity; delivery complexity	[235,236]
Exosome-Based	STX-S + N	Spike + Nucleocapsid	Delivers antigens via engineered exosomes mimicking natural presentation	Engineered extracellular vesicles	Strong CD8^+^ T cell and antibody responses in preclinical models	Not fully reported	Clinical efficacy and scalability yet unknown	[237,238]
Virus-Like Particles (VLPs)	CoVLP + AS03	Spike	Presents spike protein in repetitive arrays to stimulate B cells	Protein-based with adjuvant	~70–80% efficacy against ancestral strains (Phase III)	Fatigue, injection site pain; mild systemic symptoms	Cold-chain requirements and manufacturing cost	[239,240]
DNA-Based	ZyCoV-D	Spike	DNA plasmid is transcribed in host cells to produce viral antigen	Needle-free intradermal jet	~67% efficacy; moderate antibody response (India trials)	Injection site swelling, fatigue	Lower immunogenicity; boosters often needed	[241,242,243]
Recombinant Protein-Based	Novavax NVX-CoV2373	Spike	Protein subunit with adjuvant elicits targeted immune response	Protein subunit with Matrix-M adjuvant	High efficacy against symptomatic COVID-19 in trials	Fatigue, headache, local/injection site pain	Multiple dose requirement; logistical hurdles	[244,245]
Inactivated Virus	Covaxin, Sinopharm, Sinovac, BBV152	Whole virus	Chemically inactivated virus triggers broad immune response	Inactivated virus with adjuvant	Moderate protection against severe disease	Fever, fatigue, injection site pain	Requires multiple doses; lower immunogenicity	[246,247]
Universal Coronavirus Vaccines	Mosaic nanoparticles, pan-sarbecovirus platforms	Conserved epitopes from multiple strains	Designed to induce broad immunity against variants and future coronaviruses	mRNA, protein nanoparticle, viral vector	Broad cross-reactive immunity in animal studies	Mild local/systemic effects in preclinical studies	No licensed product yet; efficacy under investigation	[248,249]
